# Linking extreme light availability to cellular function in algae-dominated communities on the Greenland Ice Sheet

**DOI:** 10.1093/femsec/fiaf095

**Published:** 2025-09-27

**Authors:** Helen K Feord, Christoph Keuschnig, Christopher B Trivedi, Rey Mourot, Athanasios Zervas, Thomas Turpin-Jelfs, Martyn Tranter, Alexandre M Anesio, Lorenz Adrian, Liane G Benning

**Affiliations:** Interface Geochemistry, GFZ Helmholtz Center for Geosciences, 14473 Potsdam, Germany; Interface Geochemistry, GFZ Helmholtz Center for Geosciences, 14473 Potsdam, Germany; Interface Geochemistry, GFZ Helmholtz Center for Geosciences, 14473 Potsdam, Germany; Interface Geochemistry, GFZ Helmholtz Center for Geosciences, 14473 Potsdam, Germany; Department of Earth Sciences, Freie Universität Berlin, 12249 Berlin, Germany; Aix Marseille Université, Université de Toulon, CNRS, IRD, MIO, 13009 Marseille, France; Department of Environmental Science, Aarhus University, 4000 Roskilde, Denmark; Department of Environmental Science, Aarhus University, 4000 Roskilde, Denmark; Department of Environmental Science, Aarhus University, 4000 Roskilde, Denmark; Department of Environmental Science, Aarhus University, 4000 Roskilde, Denmark; Department Molecular Environmental Biotechnology, Helmholtz Centre for Environmental Research - UFZ, 04318 Leipzig, Germany; Chair of Geobiotechnology, Technische Universität Berlin, 10623 Berlin, Germany; Interface Geochemistry, GFZ Helmholtz Center for Geosciences, 14473 Potsdam, Germany; Department of Earth Sciences, Freie Universität Berlin, 12249 Berlin, Germany

**Keywords:** Dark adaptation, Glacier Ice algae, Greenland Ice Sheet (GrIS), High light survival, Metaproteomics, Metatranscriptomics

## Abstract

Glacier ice algae of the streptophyte genus *Ancylonema* bloom on glaciers globally, including the Greenland Ice Sheet. These algae survive under extreme high light conditions in the summer, as well as under very low light or total darkness during (polar) winters and winter burial under snow. However, little is known about the cellular mechanisms underpinning glacier ice algae ecophysiological plasticity in response to extreme light availability. To address this knowledge gap, we evaluated the response of *Ancylonema*-dominated taxa in samples from the Greenland Ice Sheet to light and dark conditions during a 12-day period using combined multi-omics analyses. The microbial community was not substantially altered during the 12 days of dark incubation, however transcriptomic analysis demonstrated that the algae-associated heterotrophs became more active in the dark. In contrast, we identified a striking algal transcriptome stability in light conditions, in addition to high oxidative stress responses and evidence for high photosystem protein turnover. We also identified transcriptional reprogramming linked to sugar uptake and phytohormone signalling during dark incubation. These results provide crucial clues into the ability of glacier ice algae to adapt and survive in a harsh and extremely variable light environment.

## Teaser

Transcriptomic and proteomic responses of glacier ice algae to extreme changes in light availability on the Greenland Ice Sheet, linked, for example, to oxidative stress, high protein turnover, and phytohormones.

## Introduction

Streptophyte algae of the genus *Ancylonema* bloom each summer on ice surfaces globally, including the Greenland Ice Sheet (GrIS; Lutz et al. 2018). These photosynthetic species possess a distinct color due to their purple/brown photoprotective pigment, purpurogallin, which in turn darkens the ice (Remias et al. 2012, Williamson et al. 2020, Halbach et al. 2022). These algae grow through the summer melt months, reducing surface albedo (Chevrollier et al. 2023). *Ancylonema* are the main primary producers on ice surfaces (Stibal et al. 2007, Yallop et al. 2012), supporting a microbial community that also includes fungi (Perini et al. 2019, 2023, Jaarsma et al. 2023), bacteria, other protists (Jaarsma et al. 2023), and viruses (Perini et al. 2024). *Ancylonema* cells survive and thrive at extreme high light intensities (for example photosynthetic active radiation above 1600 µmol photons·m^−2^·s^−1^), low temperatures, and highly oligotrophic conditions (Anesio et al. 2017, Williamson et al. 2020, McCutcheon et al. 2021, Halbach et al. 2023). In polar regions, such as on the GrIS, *Ancylonema* species also experience extreme photoperiods, with extended periods of constant or near-constant daylight in the summer, as well as total darkness through the polar winter. In both polar and alpine regions, algae are also buried under snow in the winter months, significantly reducing the sunlight they receive.

The functional responses of algae to extreme and seasonal fluctuations in light availability have revealed different survival strategies to high light and dark conditions. Response to and survival under high light has been linked to non-photochemical quenching and Light-Harvesting Complex (LHC) proteins, phytohormone signalling, oxidative stress and important retrograde chloroplast signalling, and proteolysis in other streptophyte algae (De Vries et al. 2018, Serrano-Pérez et al. 2022, Dadras et al. 2023). Photoprotective pigments have also been reported to be important for the survival of various algal taxa in high light conditions, including marine diatoms (Mangoni et al. 2009), terrestrial snow algae (Leya et al. 2009, Lutz et al. 2016), and for *Ancylonema* species (Remias et al. 2012, Williamson et al. 2020, Halbach et al. 2022). In contrast, survival under prolonged darkness for polar algae (for example diatom and kelp species) has been linked to the use of energy stores such as lipids (Handy et al. 2024), the transition into resting, hypometabolic cell stages (Joli et al. 2024), for example, with the reduction of cellular ATP (Kennedy et al. 2019) or transcription to conserve energy (Wutkowska et al. 2023), as well as employing facultative heterotrophy or autophagy (McMinn and Martin 2013, Kvernvik et al. 2018, Joli et al. 2024).

It is essential to assess how *Ancylonema* species sense and respond to changing light availability to understand the unique adaptations of this genus to glacier surfaces. However, we do not know what combination of strategies this genus uses to both bloom under intense light in the summer and how they prepare for survival under constant darkness. We addressed this knowledge gap by undertaking a 12-day incubation with *Ancylonema*-dominated communities from the GrIS, exposed to the intense light conditions of the summer months and to continuous darkness, investigating the differing changes in transcripts and proteins. We hypothesised that functional profiles in the light would be linked to photoprotection, photosynthesis, and metabolic pathways underlying cellular and community growth. In contrast, we hypothesised that, in the dark, functional profiles would be associated with a slowing of metabolism.

## Methods

### Field site and incubation set-up

**Figure 1.**
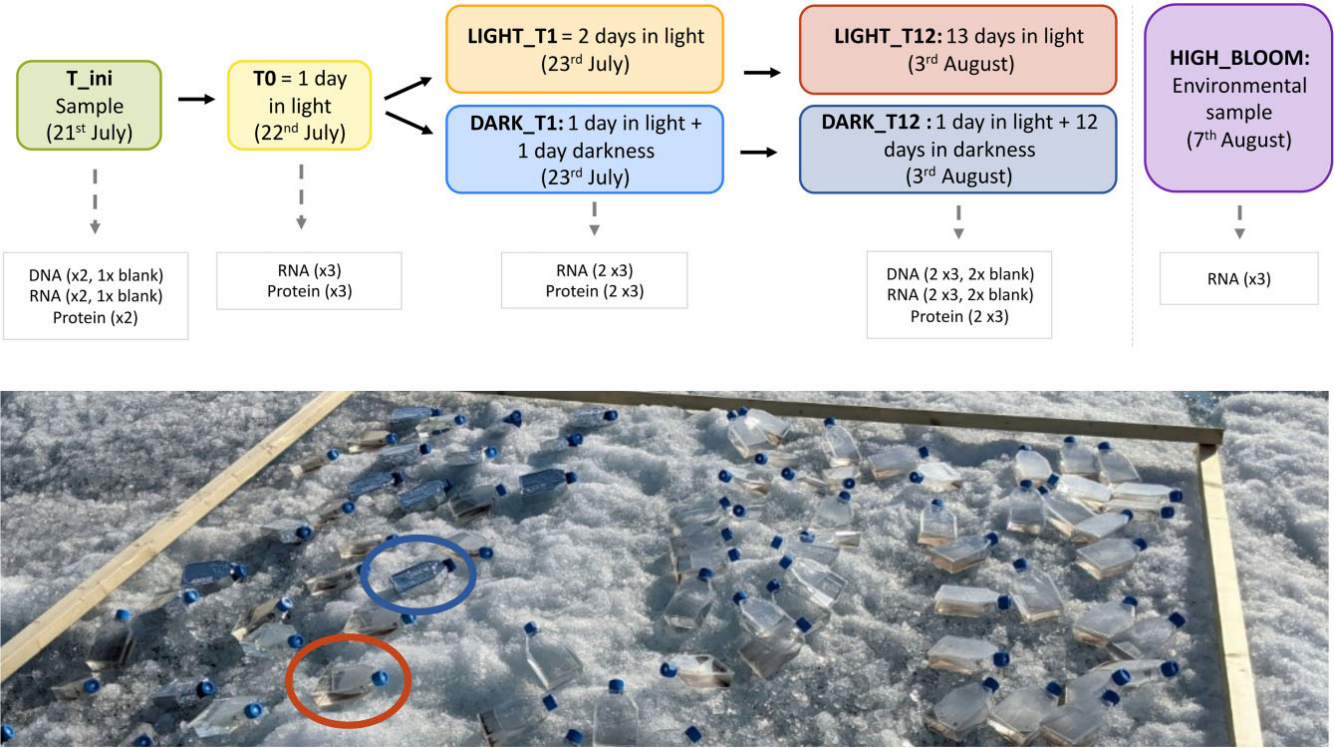
Incubation on the Greenland Ice Sheet. (A) Incubation design and analyses performed at each time step; (B) set-up for the incubation bottles directly on the bare ice surface at the DeepPurple ice camp (Fig. S1A). Example of an incubation bottle exposed to the light is shown with an orange circle, and an example of an incubation bottle exposed to the dark is shown with a blue circle. Information regarding the weather conditions in the location of the ice camp where the incubation was undertaken is available in Fig. S1B-D.

High algal biomass surface ice samples were collected in four 6.8 L Whirl-Pak^®^ bags on July 17, 2021 in south Greenland near Narsarsuaq (61°05′ N, 46°50′ W, Fig. 1A, Fig. S1A) as part of the DeepPurple ERC (https://www.deeppurple-ercsyg.eu/about) ice camp DP21 (Chevrollier et al. 2023, Jaarsma et al. 2023, Doting et al. 2024, Jensen et al. 2024, Peter et al. 2024, Rossel et al. 2025). The ice was melted over four days at ambient temperature in the field laboratory tent. Prior to experimental use, on July 21, the melted samples were thoroughly mixed by hand agitation in the Whirl-Pak bags, and 250 ml aliquots (T_ini, Fig. 1A) were filtered through single-use sterile 0.2 µm cellulose nitrate filters (Sartorius, Germany) using an EZ-stream pump and EZ-Fit manifold (Merck Millipore, USA), with the filters (and associated retained particulates) later used for DNA, RNA, and protein extractions. Aliquots were filtered in duplicate for each analysis (n = 2 for DNA, n = 2 for RNA, and n = 2 for protein). The remaining melted ice sample was distributed as 200 ml aliquots in 36 T-75 vented plastic incubation Falcon flasks (Corning, USA) that were placed on the ice surface (Fig. 1B). After 24 h (July 22) of acclimatization to ambient light (Fig. 1A), three flasks were filtered for RNA and three for protein analyses (T0). Then, half of the remaining flasks (15 flasks) were covered with aluminium foil to mimic dark conditions and all were placed back on the ice, alongside the 15 remaining vented incubation flasks left exposed to full light (Fig. 1). After a further 24 h (T1) on July 23, six light (LIGHT_T1) and six dark (DARK_T1) vented incubation flasks were removed from the ice and immediately filtered for RNA and proteins (n = 3 for dark samples and n = 3 for light for both analyses; Fig. 1A). The last time point of the experiments was after another 11 days (LIGHT_T12 and DARK_T12, August 3), when all remaining incubation flasks were filtered for DNA, RNA, and protein (n = 3 for light samples and n = 3 for dark samples for each analysis; Fig. 1A). All filtering was undertaken ∼16h00 each day to minimize diurnal variation in gene expression. For sequencing blanks (DNA and RNA) at T_ini, we filtered 250 ml of pre-filtered melted ice for each analysis. Four incubation bottles were also filled with 200 ml of the same pre-filtered melted ice, and also placed on the ice at T_ini: two were covered in aluminium foil at T0, for light and dark blanks (n = 1 for light and n = 1 for dark for both DNA and RNA) at T12 (Fig. 1A). Later in the 2021 field season (August 7), we collected a further three replicates of surface ice with high glacier ice algae biomass for RNA sequencing (HIGH_BLOOM, Fig. 1A). After filtering, all samples for DNA, RNA, and protein were kept frozen at -80°C in a portable freezer (Sterling Ultracold, USA). Samples were transported back to Germany in a liquid nitrogen-cooled cryoshipper and stored at -80°C until further processing. To assess the variability in environmental conditions during the incubation, air temperature, short wave incoming radiation, and cloud cover data were retrieved from the nearby (600 m) QAS-M weather station (https://promice.org/ ;  Fig. S1B-D).

### DNA extraction, sequencing, and analysis

DNA extraction, amplicon sequencing, and analysis were undertaken with the same workflow as previously published by Peter et al. (2024). DNA was extracted with a PowerSoil Pro kit (QIAGEN, Germany). Prokaryotic 16*S* rRNA primers (Bakt_341F CCTACGGGNGGCWGCAG and Bakt_805R GACTACHVGGGTATCTAATCC) and eukaryotic *18S* rRNA primers (528F GCGGTAATTCCAGCTCCAA and 706R AATCCRAGAATTTCACCTCT) were used to amplify DNA. DNA was sequenced on an Illumina MiSeq using the V2 kit (Illumina Inc., USA; (Cheung et al. 2010, Herlemann et al. 2011). Raw sequences were pre-processed and analysed using the DADA2 package v. 1.16 in R v. 4.2.2 for marker-gene analysis (Callahan et al. 2016, R Core Team 2023). Sequence taxonomy was assigned with SILVA v138 database (Abarenkov et al. 2024). The taxonomic assignment for the 100 most abundant 18*S* rRNA ASVs was done by hand using NCBI BLASTn (https://blast.ncbi.nlm.nih.gov/Blast.cgi)

### RNA extraction, sequencing, and analysis

Total RNA was extracted from frozen filters (Fig. 1A) using a RNeasy PowerSoil total RNA extraction kit (QIAGEN) following the manufacturer’s instructions. RNA was quantified on a Qubit 4 device (Thermo Fisher Scientific, USA) using the High-Sensitivity RNA assay. Residual DNA was removed with the DNAse Max kit (QIAGEN) following the manufacturer’s instructions. The polyadenylated mRNA was extracted from the samples using the Poly(A) RNA Selection Kit v1.5 (SKU 157.96, LEXOGEN, Austria) prior to building sequencing libraries using the CORALL Total-RNA-Seq v2 kit (SKU 171.24, LEXOGEN, Austria) following the manufacturer’s instructions. RNA concentrations in the resulting libraries were quantified on a Qubit 4 device using the 1 x dsDNA High Sensitivity Assay (Thermo Scientific) and the size distribution in the RNA preparations were visualized on a Tapestation 4150 using the D1000 Screentape and Reagents (Agilent Technologies, Germany). The libraries were pooled equimolarly and sequenced on a NextSeq 500 (Illumina) using the 300 cycles (151 bp pair-end mode) v2.5 chemistry kit. Ribosomal RNA reads were removed using RiboDetector (Deng et al. 2022) and remaining reads were assembled using Trinity (Haas et al. 2013). Dereplication at 100% was performed to remove identical sequences using cd-hit to avoid bias in count data in downstream analysis. Open reading frames (ORFs) were determined with Prodigal (Hyatt et al. 2010) using the Anvi’o suite (Eren et al. 2021) which confirmed that ∼99% of the assembled and dereplicated sequences were coding sequences (Fig. S2A). ORFs were exported as FASTA file from the Anvi’o database and sam-files of mapped back short reads to the ORFs were generated using Bowtie2 (Langmead and Salzberg 2012). BAM-files were created using SAM tools (Danecek et al. 2021) and sorted with the anvi-init-bam command from Anvi’o. Sorted BAM-files were used to create a count table of read-counts per ORF for each sample using featureCounts (Liao et al. 2014). RNA reads from the blank samples clustered separately from all the biological samples on a PCoA, and had a very low read number, and thus were removed before downstream analysis (Fig. S2B).

### Protein extraction, digestion, nLC-MS/MS, and analysis

Protein was extracted from frozen filters using a slightly modified extraction protocol from Deusch and Seifert (2015), following the same method as Feord et al. (2025). In brief, filters were cut into small pieces using sterile scissors and tweezers, and incubated with 1 ml of a 1 mM phenylmethylsulfonyl fluoride, 50 mM, pH 7.5 Tris-HCl and 0.1 mg/mL chloramphenicol solution. Samples were vortexed and mixed with 1.5 ml of a 20 mM pH 7.5 Tris-HCl and 2% SDS solution. Samples were vortexed again and incubated at 60°C in a thermomixer (Eppendorf, Germany) for 20 min shaken at 1000 RPM. 5 ml of buffer containing 20 mM Tris-HCl pH 7.5, 0.1 mg/mL MgCl_2_. 1 mM phenylmethylsulfonyl fluoride, and 1 µg/mL DNase I was added, samples were mixed and transferred to a cold sonication bath and sonicated for 6 min. Samples were incubated in a thermomixer at 37°C shaken at 1000 RPM. Samples were centrifuged twice at 4 000 x*g* to remove cell debris, minerals, and filter pieces and the supernatant was precipitated for 20 min on ice with 20% v/v trichloroacetic acid. Protein collected by centrifugation at 12 000 x *g* (4°C) for 30 min and washed with MS-grade acetone three times at 12 000 x*g* (4°C) for 10 min. Pellets were air-dried and resuspended in 2x Laemmli buffer.

From each protein extract, 19 µL were boiled with 1 µL β*-*mercaptoethanol for 10 min at 95°C and after cooling they were loaded on an SDS-PAGE gel, run for 10 min, and gel pieces were cut out and stored at -20°C until further processing. Gel pieces were destained in Coomassie destaining solution, and washed twice with water (room temperature, RT, shaking, 5 min) and twice with 50 mM ammonium bicarbonate (Ambic) buffer (RT, shaking, 10 min). Gel pieces were further incubated in 10 mM 1,4-dithiothreitol shaking at RT for 1 h to reduce disulfide bonds, followed by 1 h incubation in 100 mM 2-iodacetamide, shaking at RT to prevent the reoxidation of cysteine SH-groups. Before digestion, gel pieces were incubated shaking at RT in acetonitrile (5 min), 50 mM Ambic buffer (10 min), and acetonitrile again (5 min). Proteins were digested into peptides by overnight in-gel digestion with trypsin (20 µL of a 5 ng/µL solution), shaking at 37°C. Peptides were extracted by incubating gel pieces with 30 µL 5 mM Ambic buffer (10 min, RT, shaking), and twice with 30 µL extraction buffer (5% formic acid, 50% acetonitrile). The combined 90 µL extracted peptides from the previous steps were dried in a vacuum centrifuge previous to zip-tipping. Peptides were zip-tipped with ZipTip_μ−C18_ (Millipore, 2 μg capacity), dried, resuspended in 0.1% formic acid, and transferred to LC-MS vials. Nano-liquid chromatography tandem mass spectrometry (nLC-MS/MS) was undertaken as previously published (Seidel et al. 2018, Soder‐Walz et al. 2023).

To identify proteins from raw spectra, Proteome Discover (v2.4.0.305, Thermo Fisher Scientific) was used, with spectra searched against a database of translated metatranscriptomes from the GrIS (Perini et al. 2024). The following settings were used: dynamic modification: oxidation (+15.995 Da (Met)), static modification: carbamidomethyl (+57.021 Da (Cys)), precursor selection: MS1 precursor (minimum precursor mass: 150 Da, max: 10,000 Da, minimum peak count: 1), Sequest HT algorithm to match PSM to peptide sequences, max equal modification per peptide: 3, max number of missed cleavages was 2, and peptides lengths between 6 and 144 amino acids were allowed. Protein group intensities were normalised to sum intensity. One sample (DARK_T12_3), had a much lower count number and sum abundance (Fig. S3A-B), and was removed for downstream statistical analysis.

### Sequence annotation

Taxonomic annotations of transcript (using translated ORFs) and protein sequences (using master proteins sequences) was undertaken as following. Sequences were analysed by BLASTp against the ncbi nr databases (downloaded 05.06.2023), with –evalue 1e-5 and –top 500 using DIAMOND v0.8.22 (Buchfink et al. 2015) and sequence taxonomy was assigned using MEGAN v6.24.23 (Huson et al. 2016), by assigning the last common ancestor of each sequence (using MEGAN’s “LCA” algorithm) with the following parameters: Min Score: 50, Max Expected: 0.0001, Min Percent Identity: 0, Top Percent Identity: 10.0, Min Support Percent: 0.01, Min Support: 0, Min read Length: 0, LCA Algorithm: weighted, Percent to Cover :80, using a February 2022 MEGAN map. Sequences were annotated with INTERPROSCAN (Hunter et al. 2009), with GO terms annotated using interpro2GO.

### Statistical data analysis

Unless otherwise stated, all analysis was undertaken using R v4.3.0 (R Core Team 2023). Differential analysis of transcripts was undertaken using DESEQ2 v1.40.2 (Love et al. 2014), retaining only transcripts for analysis that had counts above 0 in at least 4 samples to remove outliers. Transcript abundance was considered significantly different between samples if the *p*-value adjusted with FDR was ≤ 0.05, and the log2 fold change was ≥ 2. For the protein data, downstream statistical analysis, all missing values were replaced by a 0, and a Kruskal-Wallis test (*p*-value ≤ 0.05), followed by a post-hoc Dunn test (*p*-value ≤ 0.05) was used for statistical analysis with rstatix v0.7.2 (Kassambara 2023).

In order to control for any bottle effect in our analysis, we combined the statistical test results (DESEQ for the RNA data and Kruskal-Wallis test/Dunn test for the protein data) to generate the final list of differentially regulated transcripts and differentially regulated proteins. We tested for three variables (“Dark”, “Light”, and “Time” in bottles/bottle effect) across two time points (T1 and T12). Transcripts and proteins groups were only deemed up- or down-regulated in the dark at a given time (T1 or T12) if the significant trend between T0 and the dark sample (for example T0 *<* DARK_T12) also existed between the dark samples and the light sample at the same time point (for example LIGHT_T12 *<* DARK_T12). Conversely, a transcript/protein group up- or down-regulated in the light compared to T0 (for example T0 *<* LIGHT_T12), also needed to show the same significant trend compared with the dark sample at the same time point (for example LIGHT_T12 *>* DARK_T12). Finally, transcripts/protein groups showing a similar significant trend for light and dark at the same time point compared to T0 (for example if LIGHT_T12 > T0 and DARK_T12 > T0) were identified as up- and down-regulated by time in bottles. Throughout the manuscript “Time” refers to the time in bottles and is a proxy for a possible bottle effect. Therefore, throughout the manuscript, if a transcript/protein is described to be upregulated or downregulated at T1 or T12 in light, dark, or time, it had undergone two pairwise comparisons with the same outcome.

For data plotting, the following R packages were used: vegan v2.6.6 (Dixon 2003), pheatmap v1.0.12 (Kolde 2025), and ggplot2 v3.5.1 (Wickham 2011). Phylogenetic analysis of protein sequences was undertaken using MEGA v11.0.11, with sequence alignment with MUSCLE, applying the Maximum Likelihood method with 100 bootstraps (Tamura et al. 2021). PERMANOVA and PERMADISP tests were computed using the adonis() function of the vegan v2.6–4 R package (Dixon 2003).

## Results and discussion

### Bottle incubation did not alter community composition but darkness upregulated heterotrophic transcripts

**Figure 2.**
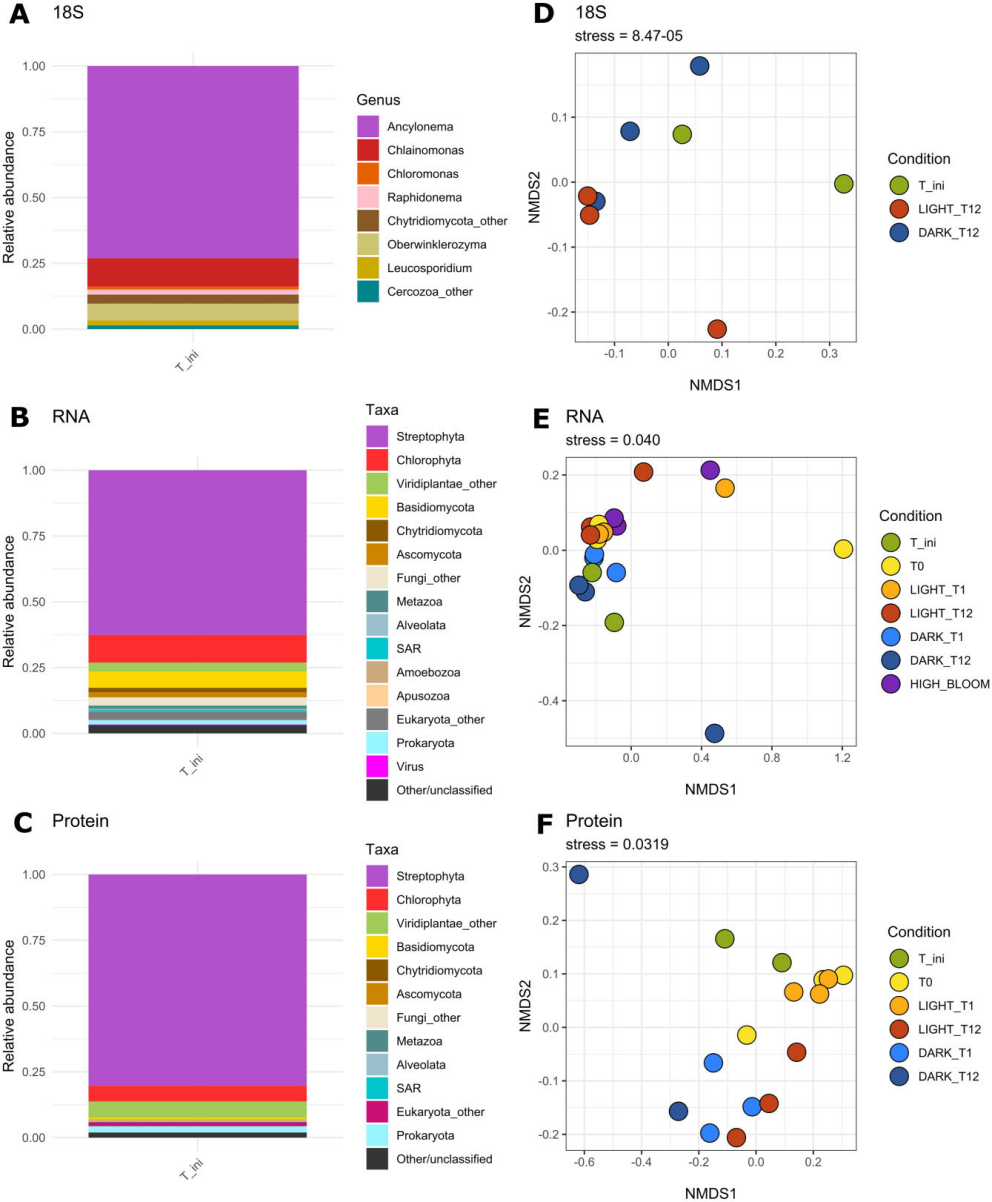
Taxonomy and sample differentiation. Relative abundance of different taxonomic groups based on (A) 18*S* gene amplicon sequencing (showing only ASVs representing over 1% of total sequences), (B) RNA (using BLASTp and MEGAN taxonomic identifications, all sequence not aligned with BLASTp were removed), and (C) protein (using BLASTp + MEGAN taxonomic identifications, all sequence not aligned with BLASTp were removed); and corresponding NMDS plots of (D) 18*S* rRNA gene amplicons, (E) RNA (using vst transformed data), and (F) protein (normalized data). The relative abundance of different taxonomic groups based on RNA of the HIGH_BLOOM sample is available in Fig. S5.

A total of 585 346 mRNA reads were assembled and mapped to ORFs (Fig. S2A), and 2 095 protein groups were quantified from the protein data (Fig. S3B). The phylum level taxonomic classification of the 18*S* rRNA gene amplicons, all transcripts, and all protein groups and their relative abundance in the initial environmental sample (T_ini; Fig. 1) was largely consistent between the three analyses (Fig. 2A-C, Fig. S4A), and matched what has previously been described for GrIS ice communities for the same site (Jaarsma et al. 2023, Doting et al. 2024, Peter et al. 2024, Rossel et al. 2025). The dominant community member in T_ini was *Ancylonema* species (identified by 18*S* sequencing). Streptophyte sequences and protein groups, assumed to belong to algae from the streptophyte *Ancylonema* genus, were also dominant in the RNA and protein data. However, the relative abundance of taxa identified with RNA suggested that the streptophyte algae were not as abundant in T_ini (∼62%), compared to later in the season as seen with the HIGH_BLOOM sample (for which ∼75% of sequences were annotated as streptophyte sequences; Fig. S5). Other algae present were various chlorophyte species (mostly *Chlainomonas*), as well as various previously identified fungal (Perini et al. 2019, 2023), protists, and bacterial species (Jaarsma et al. 2023) with viral transcripts also identified (Perini et al. 2024); Fig. 2A-C, Fig. S4). Comparison of community composition using 16*S* and 18*S* rRNA gene sequencing indicated a significant difference between initial (T_ini) and end incubation samples (LIGHT_T12, and DARK_T12; PERMANOVA *p*-value = 0.013 for 18*S* gene amplicons and p-value = 0.006 for 16*S* gene amplicons). However, we found no significant difference in pairwise comparisons between dark and light incubations (Pairwise PERMANOVA *p*-value > 0.05). PERMDISP tests also indicated no significant variability between the incubation conditions (*p*-value > 0.05; Fig. 2D, Fig. S4). It is possible that the number of replicates for this PERMANOVA test was too low to robustly identify significant differences for pairwise comparisons.

For our RNA and proteins data, T_ini samples did not cluster with the light incubation samples T0, LIGHT _T1, and LIGHT_T12 on the NMDS (Fig. 2E-F). A possible explanation for this is the four-day delay between sample collection and incubation due to slow melting in the laboratory tent away from standard high light GrIS conditions, which may have reduced cellular stress due to lower light intensity (Williamson et al. 2020, Jensen et al. 2024, Peter et al. 2024). Based on this result, only T0 was used as the starting incubation control to assess the treatment effect (dark and light) on gene expression, as we assumed T_ini samples did not represent the starting gene expression anymore. The protein NMDS also suggested that the protein profiles of LIGHT_T12 shared more similarities with the protein profiles of DARK_T1 samples (Fig. 2F), a result which was not evident for RNA (Fig. 2E). A possible explanation for this protein result could be a similarity of part of the functional response to darkness compared to the loss of external nutrient input through bottle incubation experienced in the light. For example, both treatments are known to trigger cell senescence in land plants (Buchanan‐Wollaston et al. 2005).

**Figure 3.**
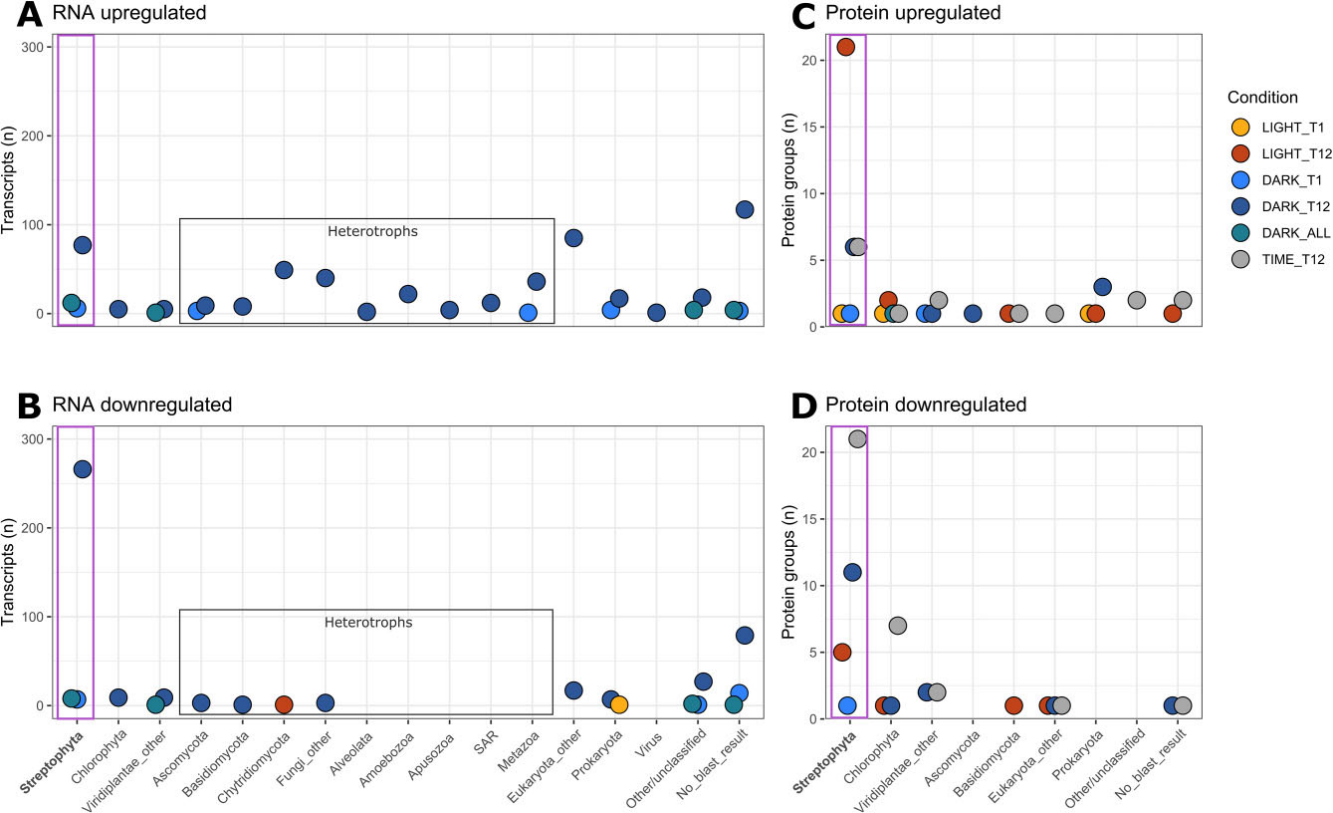
Number and taxonomy of differentially regulated transcripts (A-B) and protein groups (C-D) in the light, dark, and in time (bottle effect). Transcripts and proteins were considered up- or down-regulated in the dark (DARK_T1, DARK_T12, DARK_ALL) at a given time if the trend between T0 and the dark sample also existed between the dark samples and the light sample. The same is true for transcripts and proteins up- or down-regulated in the dark (LIGHT _T1, LIGHT_T12,), for which the trend needed to be consistent for the comparisons with T0 and the light sample at the same time point. Transcripts/protein groups showing a similar trend for light and dark at the same time point compared to T0 were identified as up- and down-regulated by time in bottles, assumed to be a bottle effect (TIME_12, see methods for more details). Transcripts and protein groups differentially regulated both at DARK_T1 and DARK_T12 are labelled as DARK_all, and transcripts and protein groups differentially regulated at LIGHT_T12 and DARK_12 (regulated in time rather than by light of dark) labelled as TIME_T12 (grey). All streptophyte transcripts and protein groups are in a purple box. Transcripts identified as eukaryotic heterotrophs are also in a grey box.

For the RNA data, the metatranscriptome appeared to have a remarkable stability in bottles in the light, suggesting a negligible bottle effect on transcripts (Fig. 3A-B, Fig. S6). 1 000 transcripts were found to be differentially regulated in dark conditions in comparison to the light incubations: a large majority (928 transcripts) were only found at DARK_T12, 33 transcripts were shared between DARK_T1 and DARK_T12 (DARK_ALL), and 39 transcripts were found to be only differentially regulated at DARK_T1 (Fig. 3A-B). Transcripts with no taxonomic classification were the most upregulated in the dark (117, Fig. 3A), followed by uncategorised eukaryotic transcripts (85 transcripts), and streptophyte transcripts (77 transcripts). At DARK_T12, an increase in transcript abundance was observed for multiple heterotrophic groups, including Chytridiomycota (49 transcripts), Metazoa (34 transcripts), and Amoeboza (22 transcripts), as well as the increase in one viral transcript (Fig. 3A). The GO terms associated with upregulated heterotrophic transcripts were indicative of growth and an increase in metabolic activity of these species (Fig. S7). The increase in Chytridiomycota species activity confirms previous suggestions that they are decomposers of GrIS algae (Perini et al. 2023). Additionally, a recent study also pointed towards an important role for viruses as a top-down control on GrIS algae (Perini et al. 2024). Most of the downregulated transcripts at DARK_T12 were streptophyte transcripts (266 transcripts; Fig. 3B). For the metaproteome data, the majority of upregulated and downregulated protein groups were annotated as streptophyte proteins (Fig. 3C-D).

**Figure 4.**
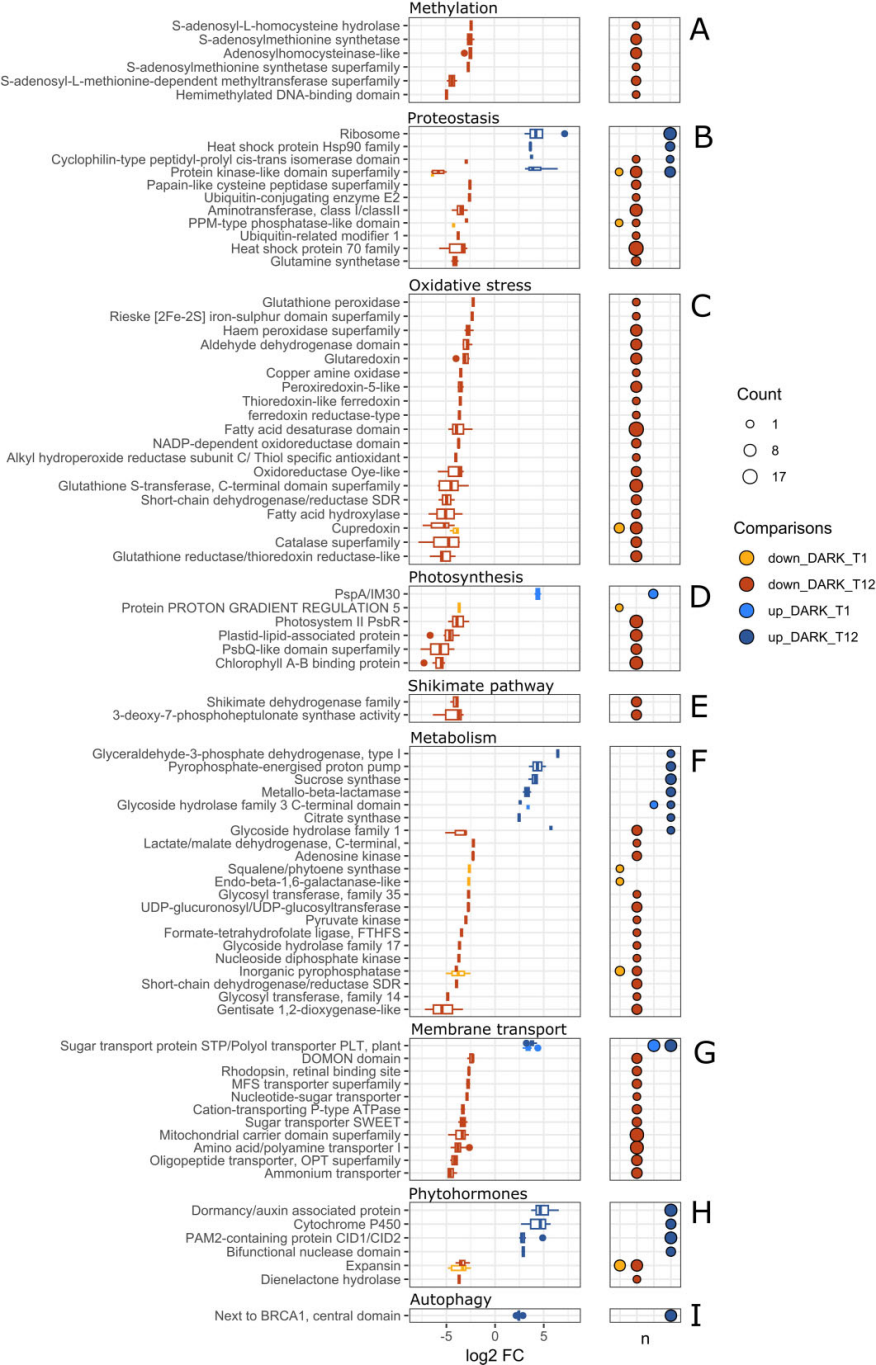
Differentially regulated streptophyte transcripts. Each transcript is plotted using one INTERPRO annotation. Transcripts with the same annotation are plotted together using a box plot (left plots). The number for each annotation shown in the right plot for each category. Transcripts were considered up- or down-regulated at in the dark at a given time if the trend between T0 and the dark sample also existed between the dark sample and the light sample at the matching timepoint (see methods for more details). The log2 fold change in abundance (log2 FC) shows the difference between light and dark at each time point (abundance change with T0 is not shown), with positive fold changes indicating upregulation in the dark and negative fold changes indicating downregulation in the dark. All transcripts with a ribosome subunit annotation are plotted here as “Ribosome”. Information on differential expression of transcripts for other taxa are available in Fig. S7 (heterotrophs) and Fig. S10 (viridiplantae other). Differentially regulated streptophyte transcripts not shown here are plotted in Fig. S8.

**Figure 5.**
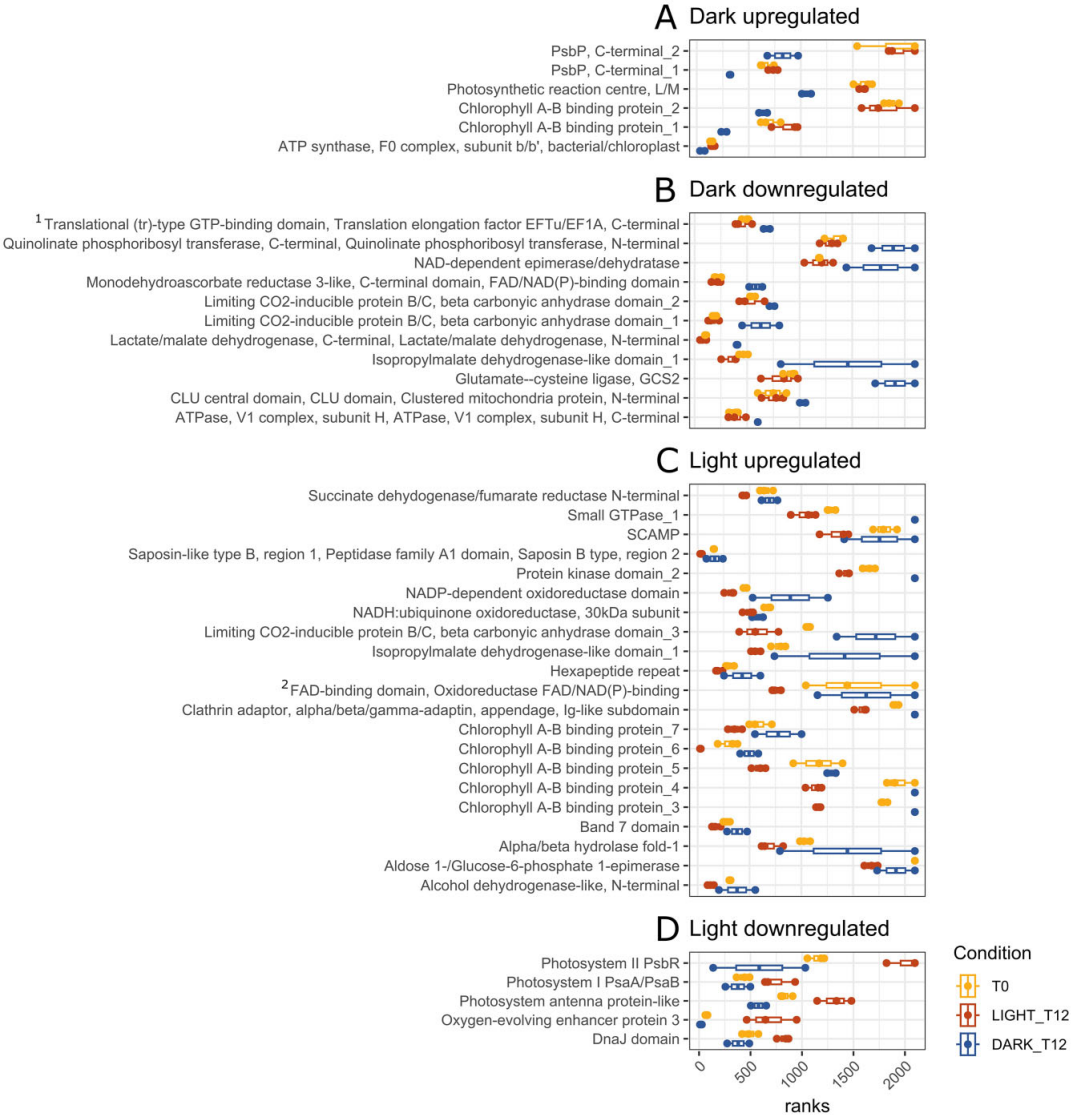
Differentially regulated streptophyte protein groups at T12. (A-B) up- and downregulated in the dark, and (C-D) up- and downregulated in the light. Proteins were considered up- or down-regulated at DARK_T12 if the trend between T0 and DARK_T12 also existed between LIGHT_T12 and DARK_12. Proteins were considered up- or down-regulated at LIGHT_T12 if the trend between T0 and LIGHT_T12 also existed between LIGHT_T12 and DARK_12 (see methods for more details). Proteins are plotted as ranks within each sample, and only T0, DARK_T12, and LIGHT_T12 are shown for figure clarity. Proteins are annotated based on PFAM domains. Two annotations are shortened on the figure, full annotations are 1. “Translation elongation factor EFTu-like, domain 2, Translational (tr)-type GTP-binding domain, Translation elongation factor EFTu/EF1A, C-terminal” and 2. “Flavoprotein pyridine nucleotide cytochrome reductase-like, FAD-binding domain, Oxidoreductase FAD/NAD(P)-binding”. Further information on the differential expression of other protein groups is available in Fig. S9.

To specifically analyse the effect of dark incubation on *Ancylonema* (Figs 4-5, Figs S8-S9), we focused on the changes in streptophyte transcript and protein group abundance for in-depth functional analysis. Information on differentially regulated transcripts and protein groups from other taxa are available in Figs S10-S11.

### Photoprotection and high protein turnover could contribute to *Ancylonema*’s survival in high light conditions

We identified significant transcriptional reprogramming of algal genes after 12 days in the dark (DARK_T12), despite little transcriptional change at DARK_T1, and no transcriptional change seen in the light conditions despite daily weather fluctuations (Fig. 3, Figs S1, S6). At day 12, we found that multiple streptophyte transcripts linked to the methyl cycle were downregulated in the dark including five S-adenosylmethionine synthetase transcripts, (Fig. 4A). This result suggests an important role for methylation in the light, as the methyl cycle is known to be important for gene expression in response to abiotic stress, in plants, for example with DNA methylation (Zhang et al. 2018). We also found multiple ribosomal transcripts upregulated in the dark (Fig. 4B): this might be necessary to support the transition from transcriptional stability to transcriptional reprogramming in the dark (Figs 2-3, Fig. S6).

In contrast to ribosomes, we found a general lowering of Heat Shock Proteins 70 transcripts (HSP70) in the dark despite an increase in ribosomes. Therefore, it is possible that this change is a result of abundant HSP70s being used as a stress response in the light, rather than the lowering of protein production in the dark. A constitutive stress response, which includes the production of heat shock proteins, has been hypothesized to be essential for the survival of the Antarctic *Chlamydomonas sp*. UWO241 (Cvetkovska et al. 2022), and could be involved in *Ancylonema* high light stress survival. Consistent with high stress in the light, we also observed a large downregulation of *Ancylonema* transcripts associated with oxidative stress, including peroxidases, in the transition from light to dark (Fig. 4C). The reduction of ROS and the concomitant increase of ROS scavenging enzymes has been documented in other dark incubations for polar and non-polar marine algae (18,62), as well as in non-photosynthetic snow bacteria in Svalbard (Sanchez-Cid et al. 2023). Furthermore, despite the production of the photoprotective purpurogallin pigment, our results suggest that *Ancylonema* cells are still experiencing significant oxidative stress under intense light conditions. This is substantiated by darkness-induced downregulation of seven transcripts with the annotation *Plastid-lipid-associated protein* (Fig. 4D). These encode for proteins which exist in the subcellular organelles plastoglobules associated with thylakoid membranes (which could be an important pool for carotenoids, such as xanthophylls) could also be linked to high light stress adaptation (Jiang et al. 2023).

A reduction of cellular ROS was also expected due to the loss of active photosynthesis in the cells during dark incubation: our RNA and protein datasets indicated a reduction of photosynthetic activity in the dark after 12 days (Figs 4-5). This included a clear downregulation of transcripts encoding Chlorophyll A-B binding proteins, PsbR proteins, and *PSQ-like domain superfamily* transcripts at DARK_T12 (Fig. 4) and the downregulation of two algal carbonic anhydrases protein groups at DARK_T12, and one upregulated at LIGHT_T12, and the upregulation of five Chlorophyll A-B binding proteins at LIGHT_T12 (Fig. 5B-C). Five photosynthesis protein groups were also reduced at LIGHT_T12 (Fig. 5D). This result could suggest increased protein degradation due to photodamage; a hypothesis further substantiated by the increase of six protein groups linked to photosynthesis at DARK_T12 (Fig. 5A), and the clear downregulation of protein degradation transcripts in the dark (Fig. 4B). *Ancylonema* cells may try to degrade and replace photodamaged photosynthesis proteins quickly with high and stable transcript production, which could ultimately lead to a net increase in such protein groups once intense light conditions have been removed. Phylogenetic analysis of the protein sequences associated with the up- and downregulated chlorophyll A-B binding transcripts and protein groups, indicated that the protein groups increased in the dark cluster with *Arabidopsis thaliana* LHC proteins (Fig. S13). Protein groups upregulated in the light and transcripts downregulated in the dark clustered with Early Light-Induced Proteins (Fig. S13), plant proteins specifically involved in photoprotection (Hutin et al. 2003). Photophysiological measurements undertaken with a 12-day dark incubation of *Ancylonema* spp.-dominated communities in a different GrIS location and year (see Supplementary Methods), confirmed that continual dark incubation in flasks alleviates cellular stress (Fig. S12, Table S1), aligning with previous reports on *Ancylonema*-dominated samples (Williamson et al. 2020, Jensen et al. 2024), and indicating that GrIS algal stress is strongly associated with high light exposure. To fully understand the contribution of high light *vs*. general light availability to cellular stress, including ROS signalling in these algal cells, further work is required to compare functional responses of *Ancylonema* under varied and controlled light conditions using laboratory cultures (Jensen et al. 2023, Remias and Procházková 2023). Five transcripts encoding early shikimate pathway enzymes were significantly lowered at DARK_T12 (Fig. 4E). The reduction of shikimate pathway enzymes could indicate a reduction in aromatic amino acid synthesis (Rieseberg et al. 2023) but also to a reduction in purpurogallin production (Bowles et al. 2024, Feord et al. 2025). If high light stress is fully removed, it would be logical for cells to reduce purpurogallin synthesis. This hypothesis is consistent with evidence of pigmentation loss for *Ancylonema* species cultivated in the laboratory under low light conditions (Jensen et al. 2023, Remias and Procházková 2023). This hypothesis is also consistent with the downregulation of three UDP glycosyltransferase transcripts which could be putatively involved in the production of purpurogallin (Fig. 4F; Mora et al. 2022).

### *Ancylonema* species transcriptional reprogramming strategies in the dark

Our metatranscriptomic and metaproteomic data indicated a slowing of cellular metabolism in the dark. This includes, for example, the downregulation of organic and inorganic membrane transport transcripts at DARK_T12, and protein groups with an *isopropylmalate dehydrogenase-like* domain, reduced at DARK_T12, which is linked to leucine synthesis (Fig. 5B-C), a clustered mitochondrial protein group, two protein groups linked to NAD production and use (Quinolinate phosphoribosyl transferase and NAD-dependent epimerase/dehydratase), and a Lactate/malate dehydrogenase protein group linked to the citric acid cycle (Fig. 5B). It is therefore likely that the cells were moving towards a hypometabolic state that could resemble cellular preparation for darkness such as during polar night or burial under snow.

Streptophyte transcriptional reprogramming in the dark indicated an important role for phytohormone signalling for *Ancylonema* species survival under variable light conditions. Phytohormones could contribute to the regulation of cell growth and survival in *Ancylonema* species cells in response to environmental stimuli, as has been identified for other streptophyte algae (Serrano-Pérez et al. 2022, Carrillo-Carrasco et al. 2025) Our transcriptomic data indicated that transcripts with the domain *dormancy/auxin associated protein*, known to be negatively regulated by auxin in land plants (Chen et al. 2023), were upregulated at DARK_T12 (Fig. 4H). Transcripts with this domain have been shown to be upregulated in dark conditions (Rae et al. 2014), can be used as a marker for dormancy, and are linked to increased stress resilience (Rae et al. 2014). Other transcripts whose expression could be tied to auxin availability were downregulated at DARK_T12: a Dienelactone hydrolase transcript (Fig. 4H; Thomas et al. 2000) and multiple expansin transcripts (Majda and Robert 2018, Serrano-Pérez et al. 2022). Auxin production has been shown to be induced under high light treatment in another streptophyte alga (Serrano-Pérez et al. 2022). Auxin treatment in the streptophyte *Penium margaritaceum* has also been shown to trigger transcriptional reprogramming and cellular proliferation (Carrillo-Carrasco et al. 2025). It is possible that high auxin production influences gene expression which contributes to high light tolerance and supports community growth in the light conditions. In contrast, a possible lowering of auxin availability in the dark could possibly halt cell division and trigger a form of cellular dormancy. Auxin has previously been quantified in the GrIS exometabolome, further underlying a putative cellular and community role for this metabolite (Doting et al. 2024).

Multiple streptophyte transcripts upregulated in the dark could also be linked to the phytohormone abscisic acid including seven transcripts with the domain *PAM2-containing protein CID1/CID2* which were upregulated at DARK_T12. This domain, present in plant transcription factors (Kariola et al. 2006), is linked to stress tolerance such as cold stress and UV-B (Pan et al. 2023). Transcripts with the domain *bifunctional nuclease domain* could also be regulated by abscisic acid availability (You et al. 2010). Both of these gene families could also be a response to increased pathogen activity (You et al. 2010, Aalto et al. 2012). Finally, the abundance of a cytochrome p450 transcript was higher a DARK_T12 (Fig. 4H): the closest plant protein, *Arabidopsis* CYP711A1, is known to be linked to the synthesis of strigolactones generally under low nutrient conditions (Sigalas et al. 2023).

Seven streptophyte transcripts, annotated with *sugar transport proteins STP/PPolyol transporter PLT, plant*, were upregulated at both DARK_T1 and DARK_T12, suggesting a transcriptomic upregulation of sugar influx, such as hexoses (Klepek et al. 2005), as an immediate and durable response to dark incubation (Fig. 4G). Alongside the increase in four sucrose synthase transcripts at DARK_T12 (Fig. 4F), this result is consistent with previous reports for a 1-week dark incubation in the arctic streptophyte *C. crenatum* (Mundt et al. 2019), for which the authors hypothesized that the algae could be surviving off organic carbon from extracellular polymeric substances (EPS) when light is absent. Starch synthases are responsible for the interconversion of sucrose with fructose and glucose and could be an indication of cells using their intracellular carbon stores in the absence of photosynthesis. Dark incubation for the angiosperm *Haberlea rhodopensis* also found an upregulation of sucrose synthase genes, alongside an increase in intracellular fructose and glucose concentrations (Durgud et al. 2018). Survival in the dark could also be linked to autophagy (Mundt et al. 2019), as six transcripts with the *Ig-like domain from next to BRCA1 gene* domain were found to be upregulated at DARK_T12 (Fig. 4I). The plant protein with this domain has a role in the mediation of autophagy under various abiotic stressors (Zhou et al. 2013).

We detailed above the first transcript and protein responses of an *Ancylonema-*dominated supraglacial microbiome to high light and continual darkness. For the dominant streptophyte glacier ice algae, we identified key processes linked to survival under intense light, including a potentially important role for methylation and the rapid turnover of photosynthetic proteins as a response to continuous photodamage. Under dark conditions, our data indicated a reduction in cellular metabolism, highlighting the possible role of phytohormone signalling for survival in darkness. While our protein data reveal the types of proteins present, we lack insights into general RNA and protein turnover rates in these cold-adapted species. Information about the turnover rates of RNA and proteins could contribute to our ability to interpret the speed of changes in metatranscriptomes and metaproteomes. Moreover, while our experimental design is similar to other studies on algal survival in polar night, including the abrupt change from light to dark (Joli et al. 2024), we also need to assess longer seasonal dynamics including the in-freezing processes and gradual rather than abrupt changes in environmental conditions to fully characterize supraglacial algal survival through the polar night.

## Supplementary Material

fiaf095_Supplemental_Files

## Data Availability

The amplicon and RNA sequencing datasets used in this study are available in the NCBI database under the BioProject PRJNA1196816. The MS protein data have been deposited to the ProteomeXchange Consortium (https://www.ebi.ac.uk/pride/) via PRIDE (identifier PXD058696).
