# Supplementary material for: Linking extreme light availability to cellular function in algae-dominated communities on the Greenland Ice Sheet
Source: FEMS Microbiol Ecol. 2025 Sep 27;101(10):fiaf095. doi: 10.1093/femsec/fiaf095 (PMC12501423; doi:10.1093/femsec/fiaf095)
Supplement: fiaf095_Supplemental_Files [file fiaf095_supplemental_files.zip › 2507_FEORD_Supplementary_material.docx]

**Supplementary material for:**

**Linking extreme light availability to cellular function in algae-dominated communities on the Greenland Ice Sheet**

Helen K. Feord^1^, Christoph Keuschnig^1^, Christopher B. Trivedi^1^, Rey Mourot^1,2,3^, Athanasios Zervas^4^, Thomas Turpin-Jelfs^4^, Martyn Tranter^4^, Alexandre M. Anesio^4^, Lorenz Adrian^5,6^, Liane G. Benning^1,2^

1. Interface Geochemistry, GFZ German Research Centre for Geosciences, Potsdam, Germany

2. Department of Earth Sciences, Freie Universität Berlin, Berlin, Germany

3. Aix Marseille Univ, Université de Toulon, CNRS, IRD, MIO, Marseille, France

4. Department of Environmental Science, Aarhus University, Roskilde, Denmark

5. Department Molecular Environmental Biotechnology, Helmholtz Centre for Environmental Research - UFZ, Leipzig, Germany

6. Chair of Geobiotechnology, Technische Universität Berlin, Berlin, Germany

**Supplementary methods: Pulse-amplitude-modulation fluorometry**

To investigate the effects of dark incubation on glacier ice algal photophysiology, an *Ancylonema*-dominated ice sample was collected on August 3, 2022, from the GrIS near Ilulissat (69°43’ N,49°86’ W). The sample was melted at ambient temperature in Whirl-Pak® bags. On August 5, the melted sample was homogenised by hand agitation, and 200 mL aliquots were distributed to six T-75 plastic vented flasks (Sarstedt, Germany) and six non-vented quartz bottles. The three plastic flasks and three quartz flasks were immediately wrapped with aluminium foil to create dark incubation conditions. All flasks were then incubated on the ice surface. Pulse-amplitude-modulation (PAM) fluorometry measurements were conducted on the initial sample (T0; August 5, 2022) and on all incubation samples after a period of 12 days (August 17, 2022).

The photophysiology of the microbial community was examined using PAM fluorometry, following methods established by Perkins *et al.* (2006). In this study, variable chlorophyll fluorescence was quantified from 3-mL samples utilizing a WaterPAM fluorometer, which was fitted with a blue emitter/detector cuvette system to facilitate continuous stirring and prevent sedimentation (Walz GmBH, Effeltrich, Germany). Prior to measurements, all samples were dark-adapted for 30 minutes. Rapid light curve (RLC) assessments involved a sequence of nine 20-second intervals during which actinic light intensities were gradually increased from 33 to 1,306 µmol photons m^−2^ s^−1^, culminating in a saturating light pulse of approximately 8,600 µmol photons m^−2^ s^−1^ for 600 milliseconds at the end of each interval. The maximum quantum efficiency (F_v_/F_m_) was calculated by determining the ratio of variable fluorescence (F_v_)—derived from the difference between the minimum (F_0_) and maximum (F_m_) fluorescence yields in the dark-adapted state—to F_m_. Moreover, the relative electron transport rate (rETR) was obtained by multiplying the quantum yield of photosystem II (Y[PSII]) with the incident light intensity, adjusted by a factor of 0.5, assuming uniform energy distribution between photosystems I and II. Theoretical maximum light utilization coefficients (α) were extracted from RLCs (rETR ∼ light intensity) analysed in R version 4.0.2, employing an iterative curve-fitting strategy adapted from Williamson et al (2020) .in accordance with the Eilers and Peeters model (Eilers and Peeters 1988). Outliers, identified as model residuals with *z*-scores exceeding ±2, were systematically excluded from the analysis.

As RLC assessments were insufficient to fully saturate PSII reaction centres within the microbial community it was not possible to derive values for rETR_max_ and E_k_ for either of the incubation experiments. However, a one-way ANOVA test followed by post-hoc Tukey tests were used to compare F_V_/F_M_ and α values between experimental groups.

References:

Eilers PHC, Peeters JCH. A model for the relationship between light intensity and the rate of photosynthesis in phytoplankton. *Ecol Model* 1988;**42**:199–215.

Perkins RG, Mouget J-L, Lefebvre S *et al.* Light response curve methodology and possible implications in the application of chlorophyll fluorescence to benthic diatoms. *Mar Biol* 2006;**149**:703–12.

Williamson CJ, Cook J, Tedstone A *et al.* Algal photophysiology drives darkening and melt of the Greenland Ice Sheet. *Proc Natl Acad Sci* 2020;**117**:5694–705.

**Supplementary Figures**


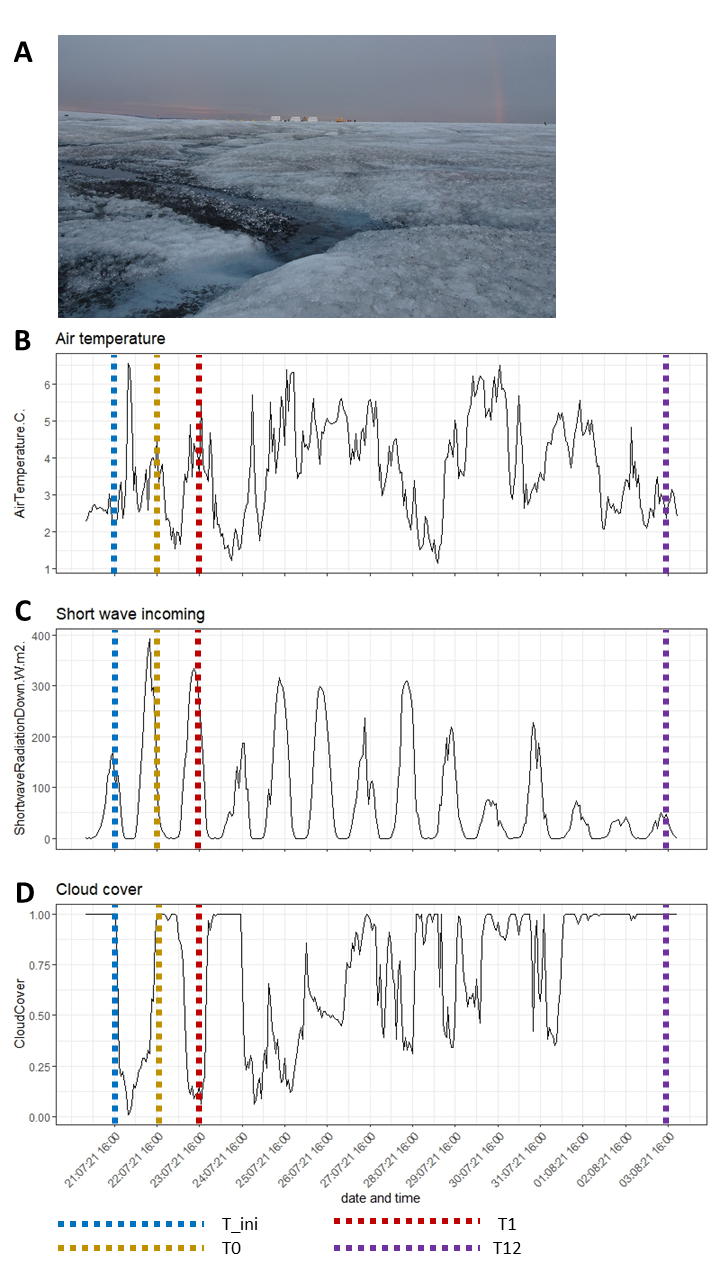
**Supplementary Figure S1: Location and weather data from the QAS_M weather station of PROMICE (**[**https://promice.org/**](https://promice.org/)**) for the duration of the incubation period**. **(A)** Representative image of the DeepPurple 2021 Ice camp on the Greenland Ice Sheet. Presented data includes **(B)** air temperature, **(C)** short wave incoming radiation (in W m^−2^), and **(D)** cloud cover. The weather for the four time points presented in this study are indicated on each graph: T_ini, T0, T1, T12.


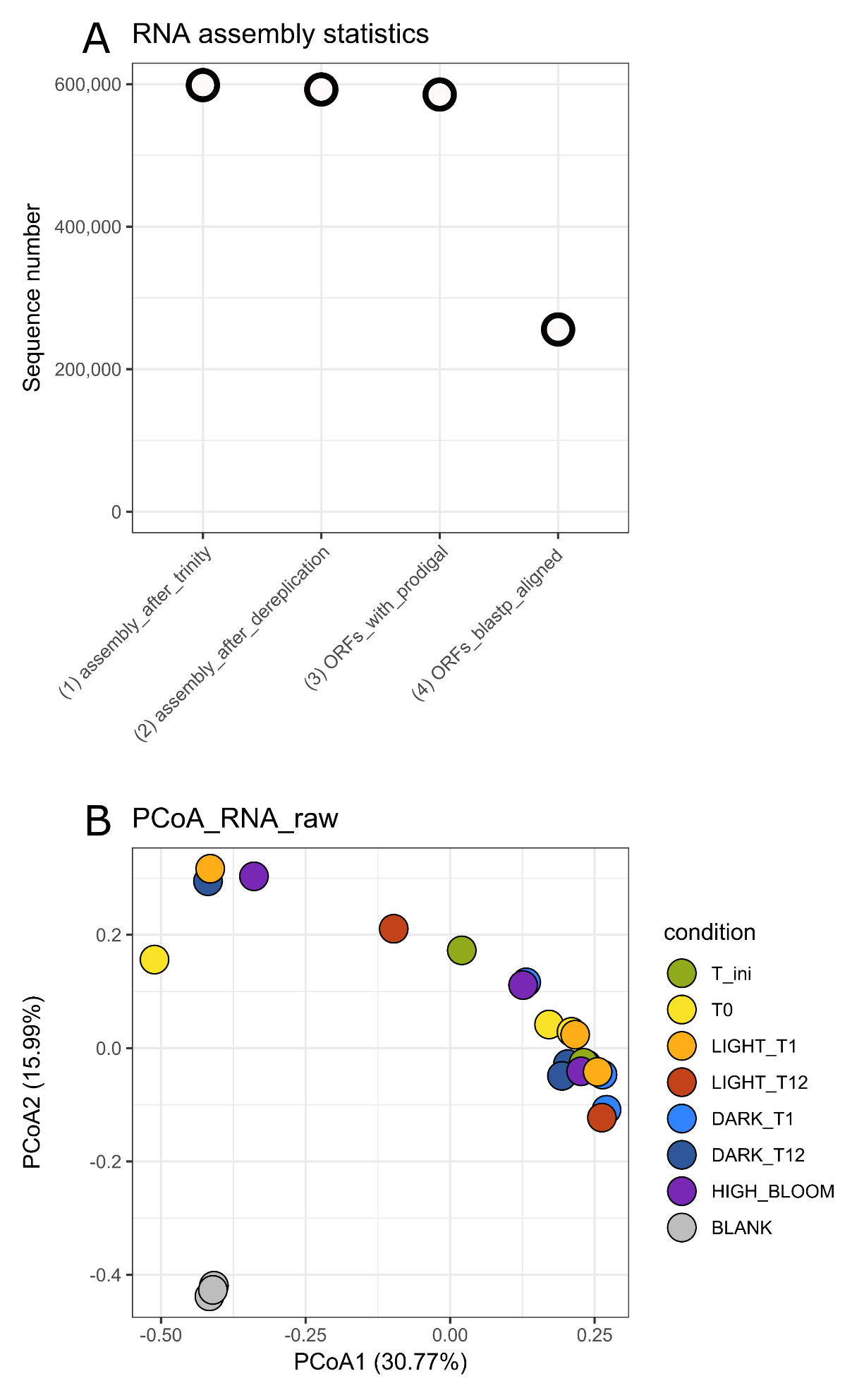


**Supplementary Figure S2: Additional information for RNA sequencing data.** **(A)** number of sequences after trinity assembly, dereplication, ORF calling with prodigal and after BLASTp searches with diamond and **(B)** PCoA figure with all raw reads including blanks.


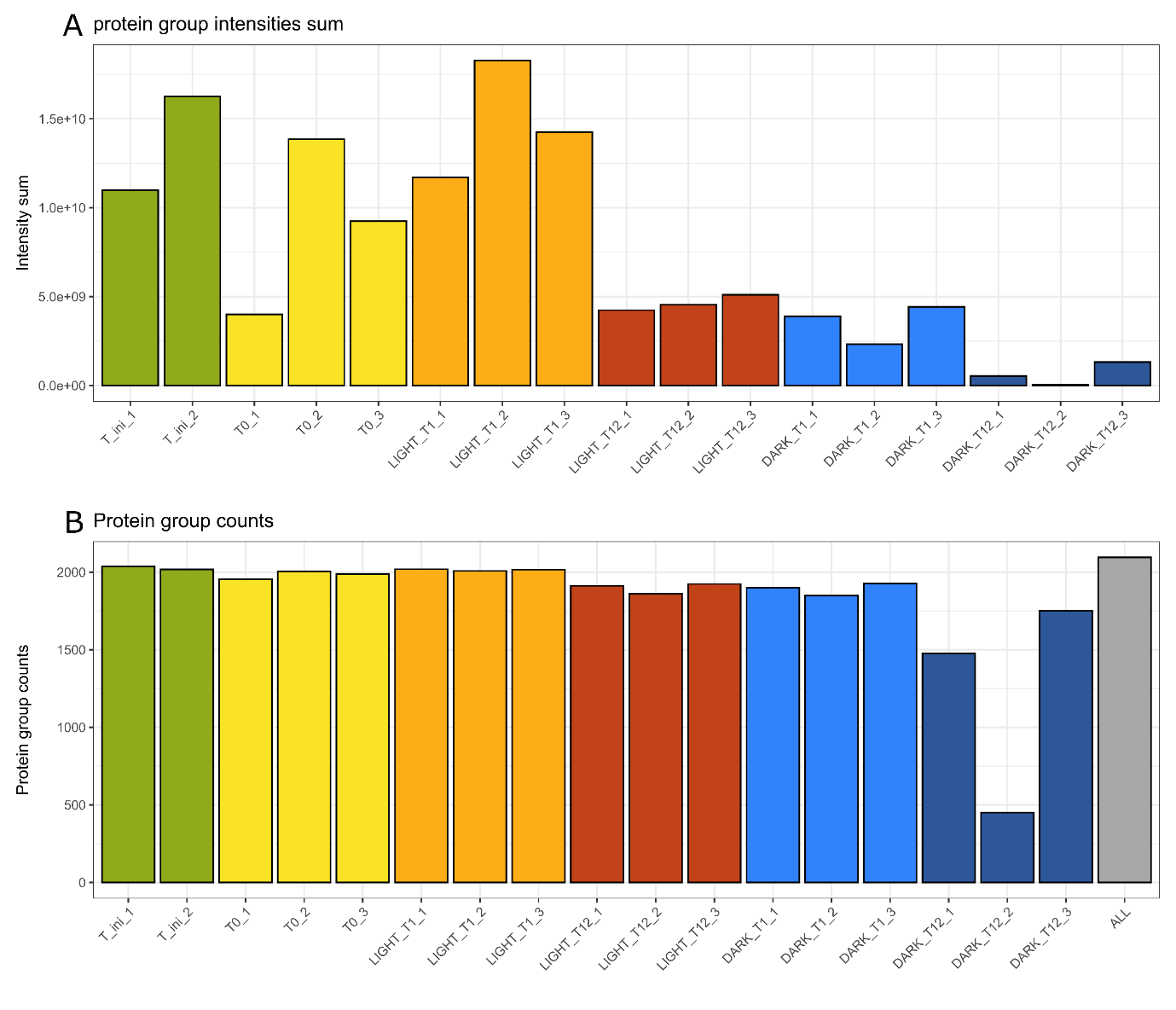


**Supplementary Figure S3: Additional information related for protein samples**. **(A)** Sum abundance of protein in each sample, and **(B)** protein group counts, including the total protein groups quantified across samples.


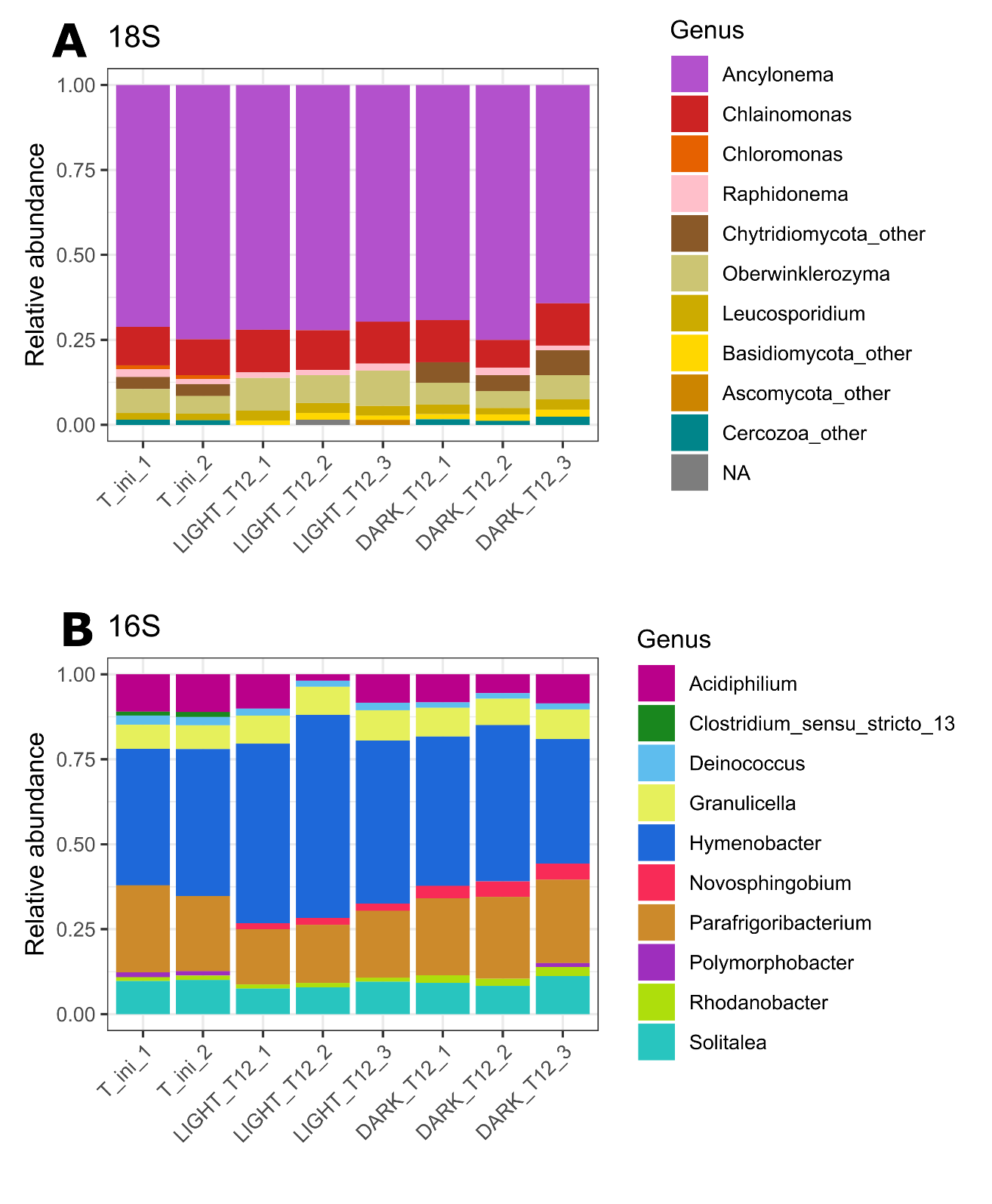


**Supplementary Figure S4: Full amplicon trends for T_ini, Light_T12, and Dark_T12.** **(A)** 18S sequencing (genus level) and **(B)** 16S sequencing (genus level). Showing taxonomic information only for OTUs representing over 1% of total sequences


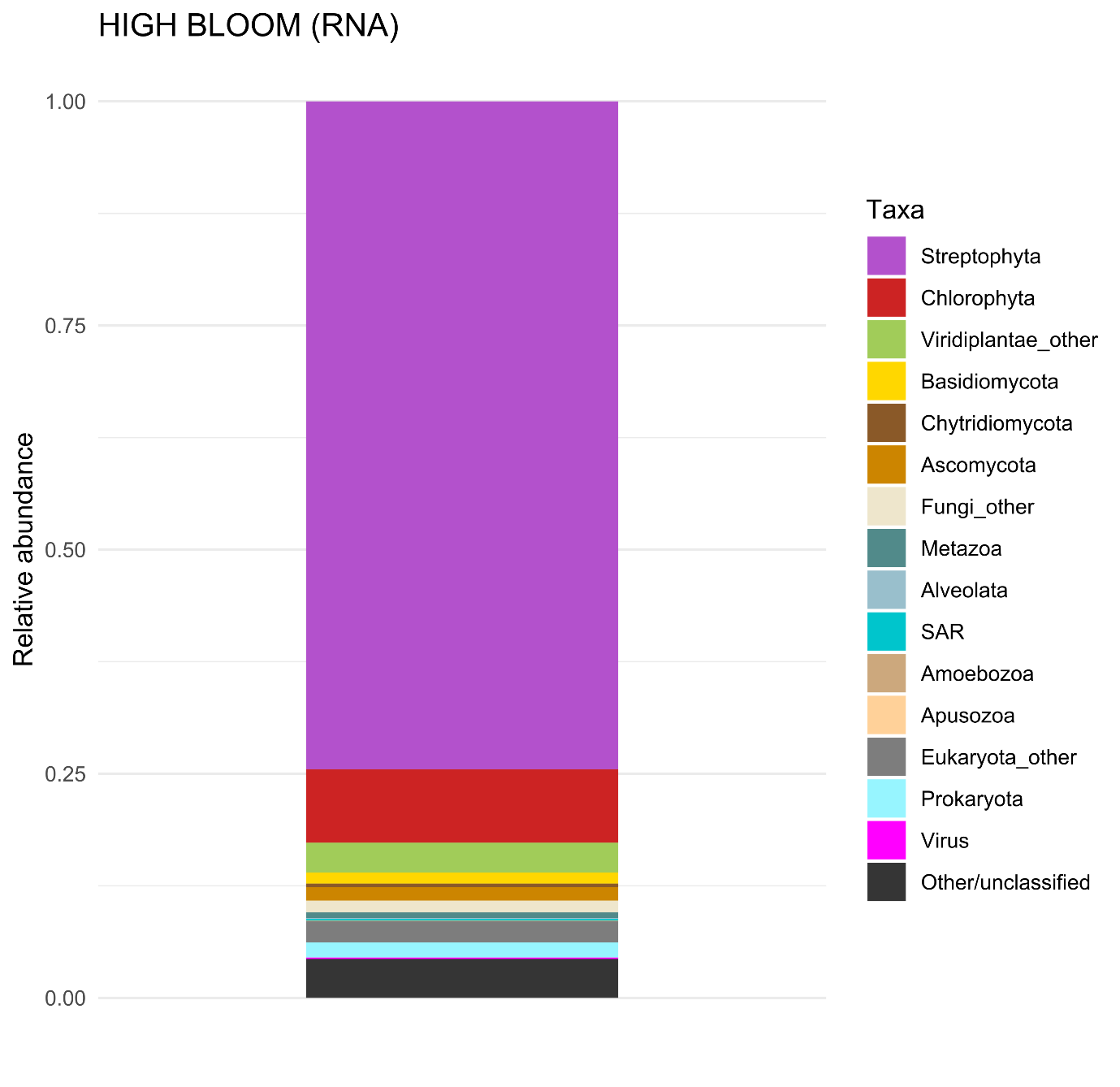


**Supplementary Figure S5: Relative abundance of taxonomic groups for the HIGH_BLOOM sample using mRNA sequences.** Taxonomy assigned using BLASTp and MEGAN, all sequence not aligned with BLASTp were removed. Data shown is the average of triplicate samples.


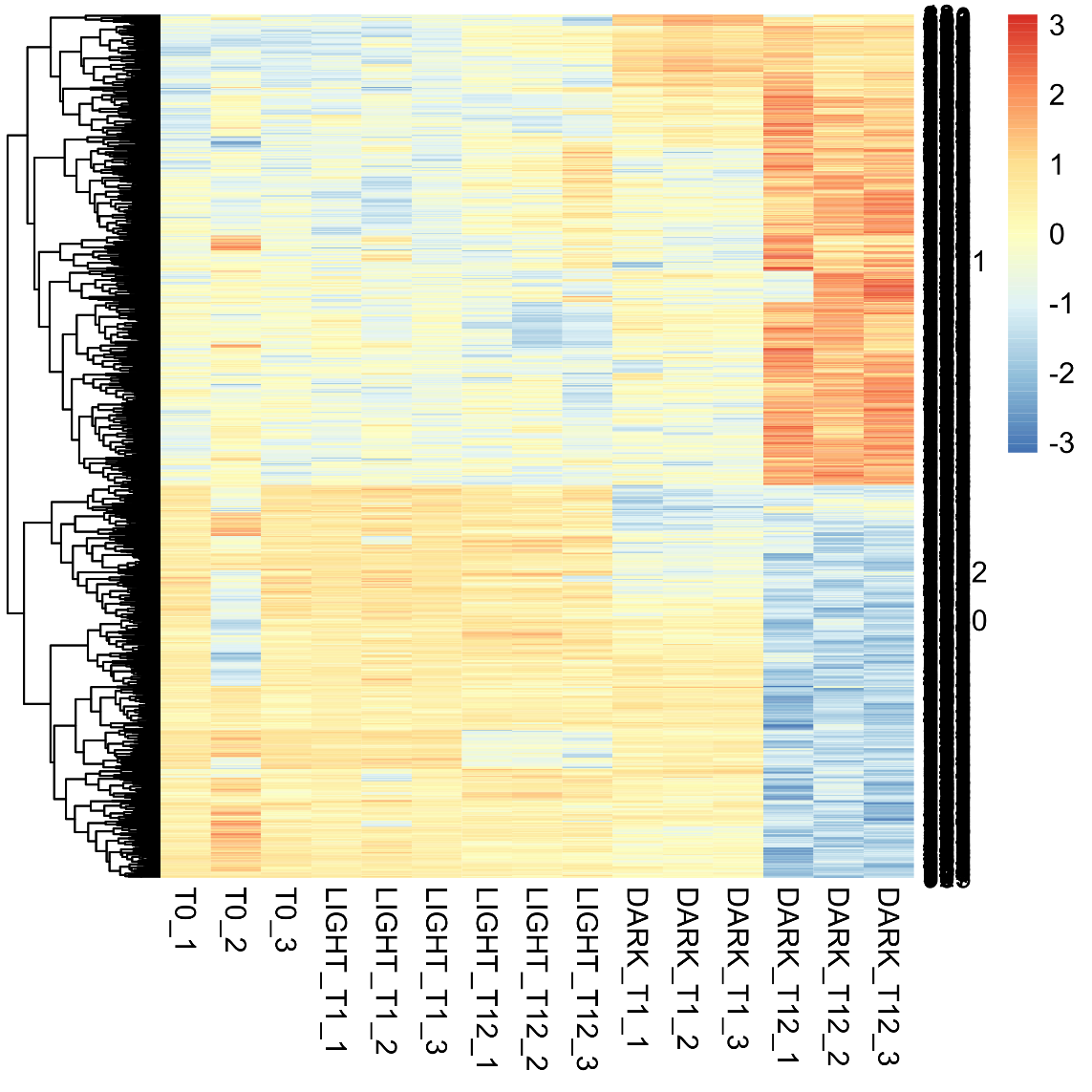


**Supplementary Figure S6: Heatmap of all differentially regulated transcripts.** Normalised data is plotted (Scaled vst()), scaled with rows, and rows are clustered with euclidean distance.


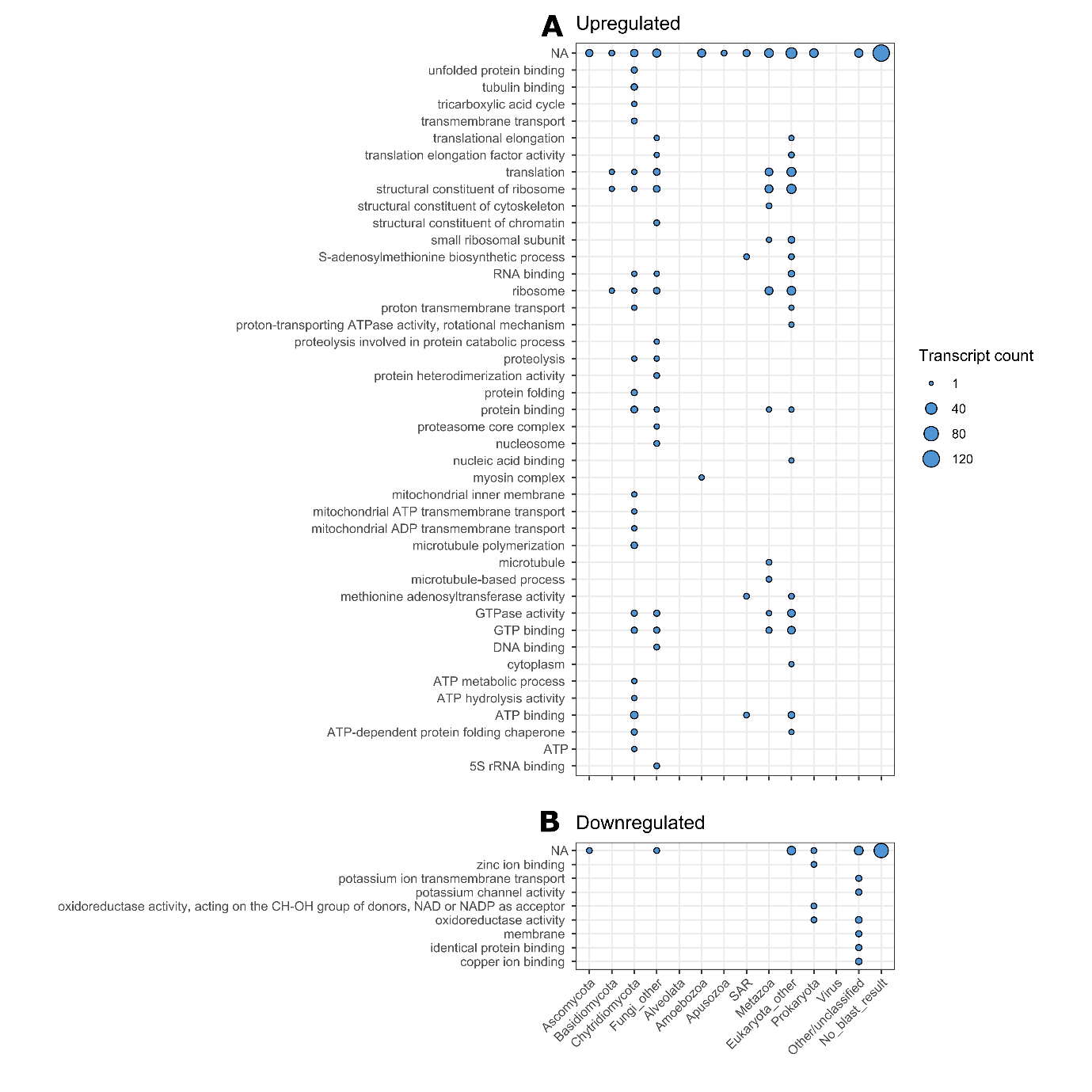


**Supplementary Figure S7: GO terms associated with up- and downregulated non-algal transcripts in the dark at DARK_T12.** Different GO terms are listed on the y axis, and taxa on the x axis. The number of transcripts associated with each taxa and GO term is shown by the size of the point.


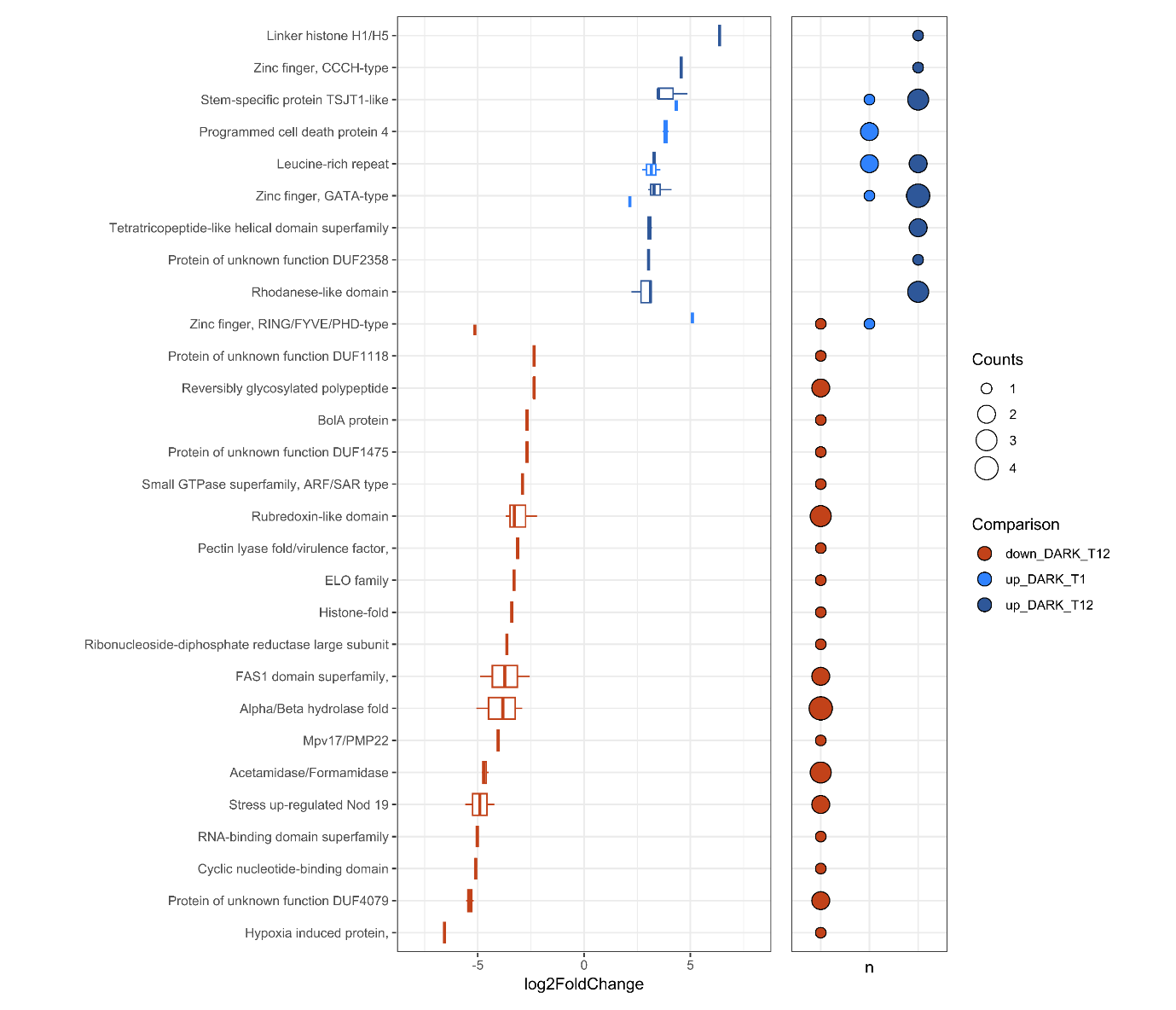


**Supplementary Figure S8: Differentially regulated streptophyte transcripts not shown in Fig. 4.**


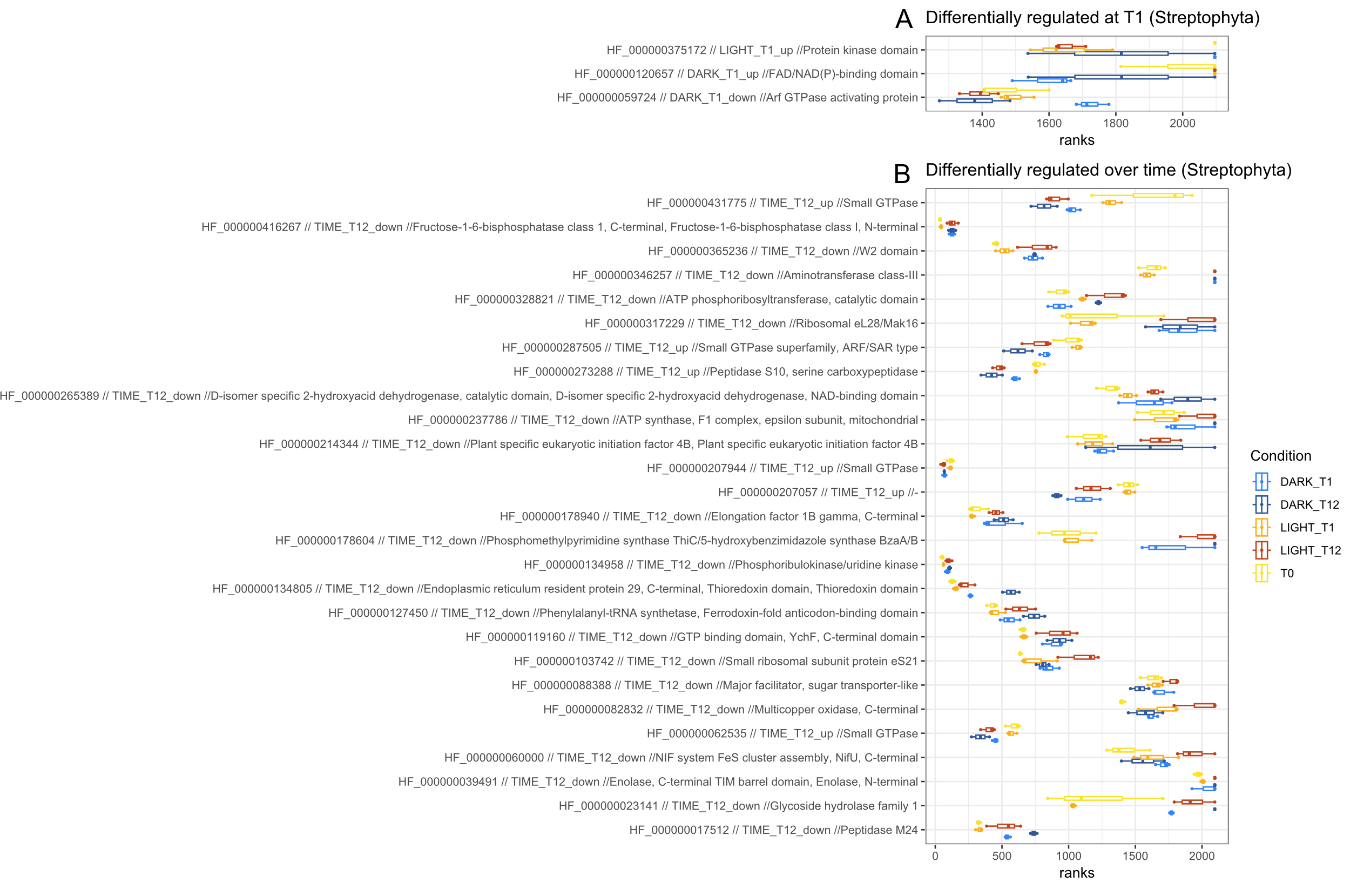


**Supplementary Figure S9: Differentially regulated Streptophyte protein groups not shown in Fig. 5**. **(A)** Streptophyte protein groups differentially regulated at T1, **(B)** Streptophyte protein groups differentially regulated over time (all at T12).


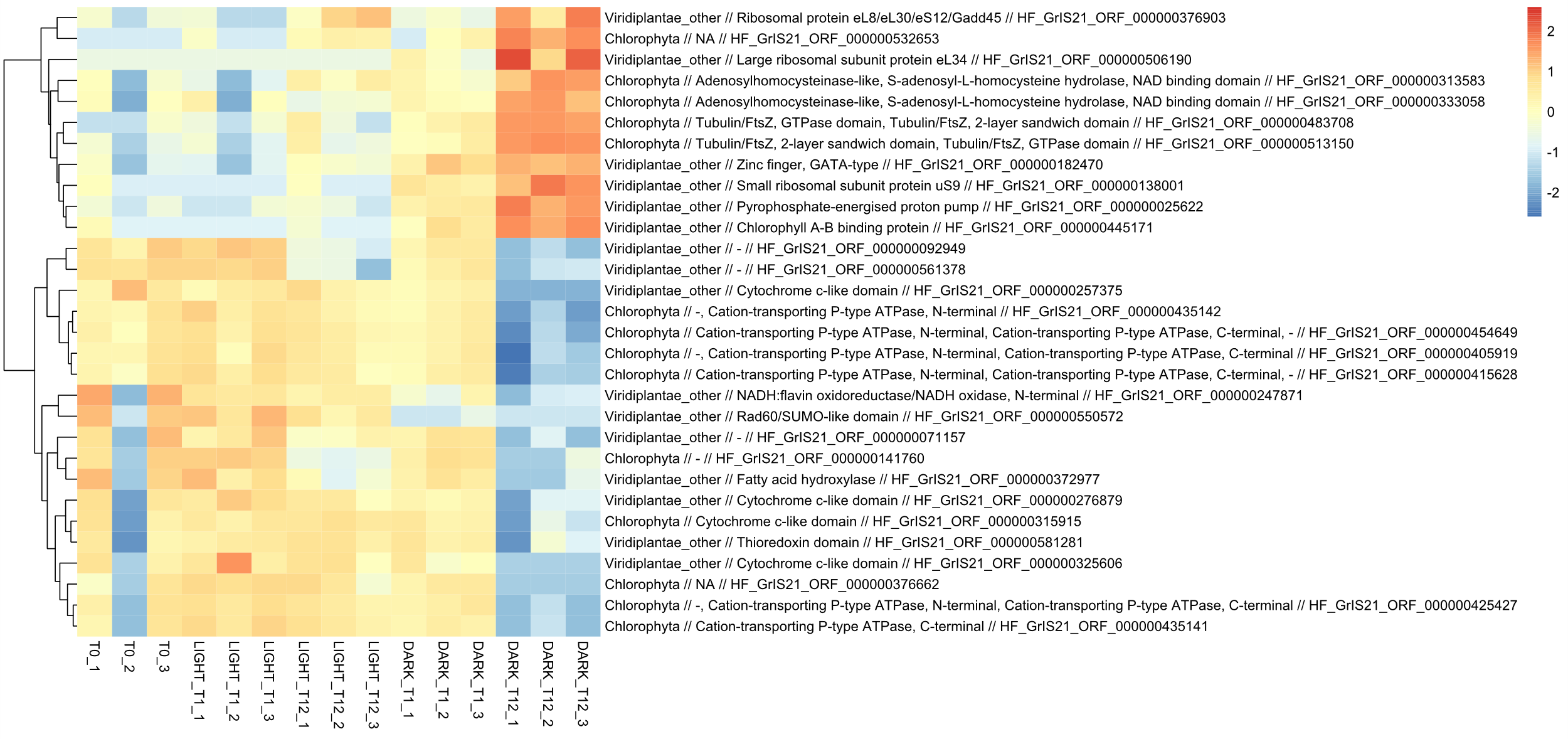


**Supplementary Figure S10: Heatmap of all non-streptophyte algal differentially regulated transcripts with all samples**. Normalised data is plotted (Scaled vst()), scaled with rows, and rows are clustered with euclidean distance.


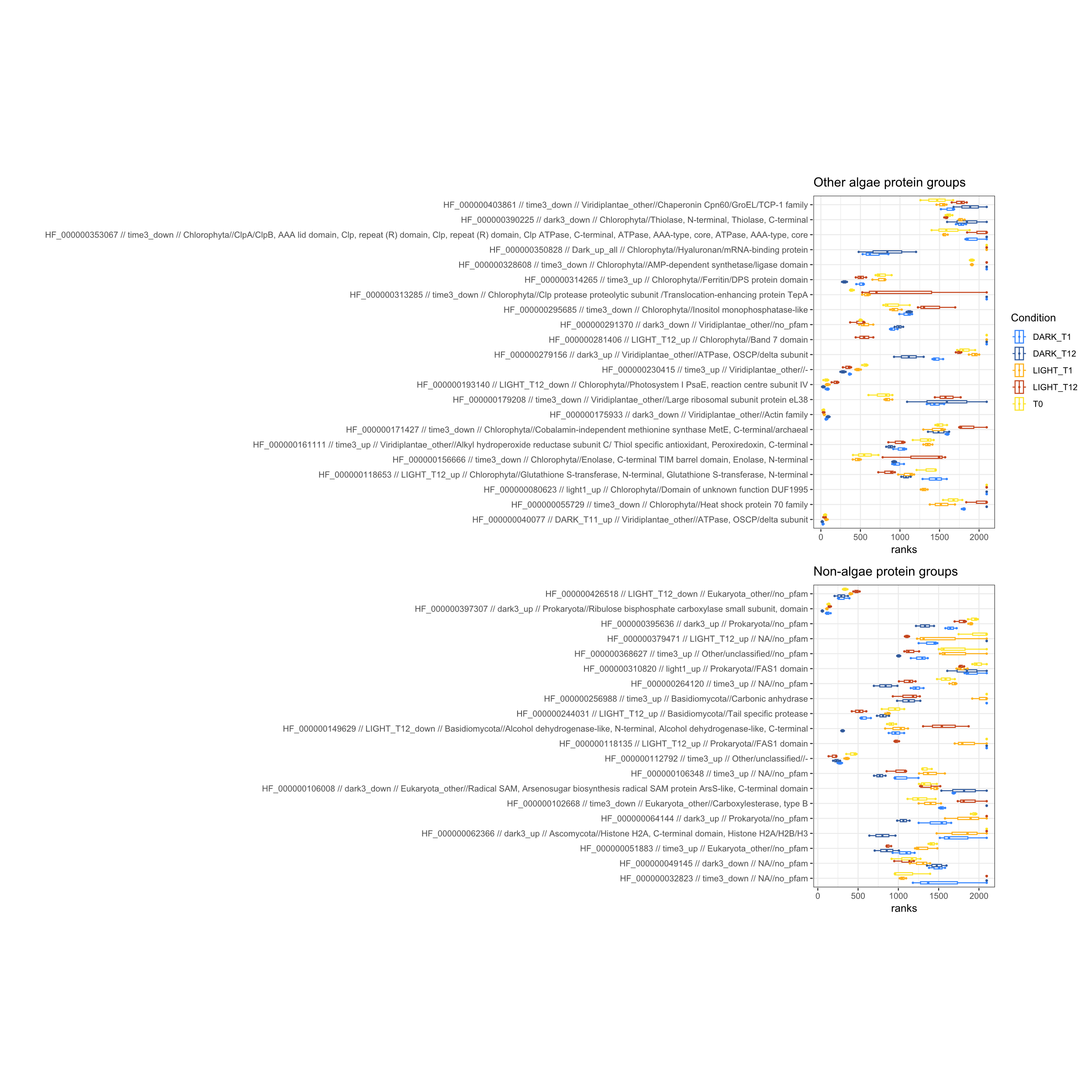


**Supplementary Figure S11: Differentially regulated non-Streptophyte protein groups not shown in Fig. 5 (A)** other algal protein groups regulated in light/dark/time, **(B)** non-algal protein groups regulated in light/dark/time. Data is plotted based on protein groups ranks in each sample. For each protein group, labelling includes accession of master protein, trend, taxa, and pfam annotation.


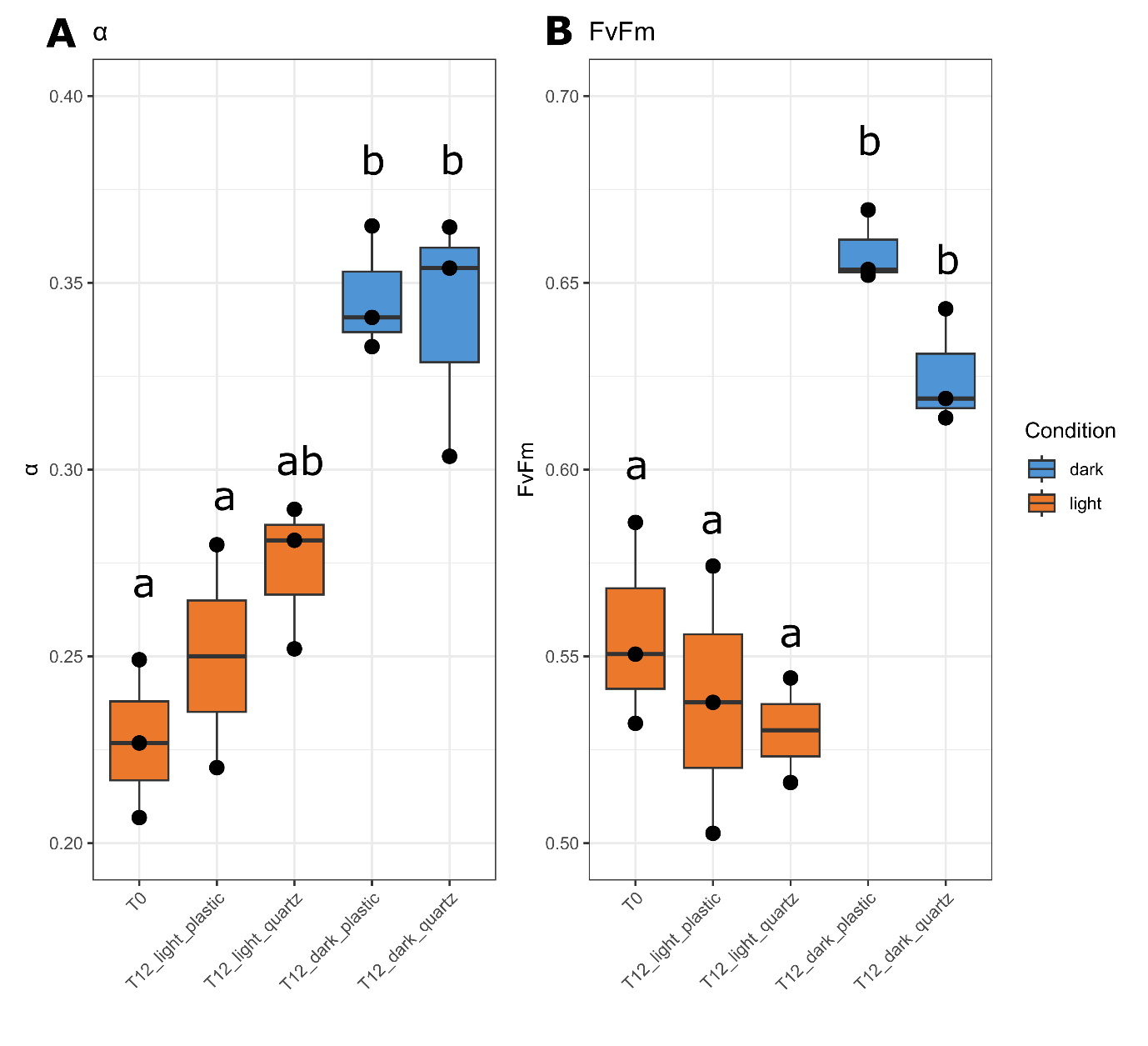
 **Supplementary Figure S12: Pulse amplitude modulation (PAM) fluorometry**. Measurements of α and F_V_/F_M_ from incubation experiments performed in 2022 (see supplementary methods), for samples incubated in plastic vented flasks and quartz bottles. Letters above box plots indicates the results of the post-hoc Tukey test showing which treatments are significantly different from each other at *p* < 0.5. Samples incubated in the dark have significantly higher α and F_V_/F_M_ values than the initial T_0_ samples and samples incubated in the light both for samples in plastic vented flasks and in quartz bottles. No significant difference was found for α and F_V_/F_M_ measurements betweeen samples incubated in plastic vented flasks compared to quartz bottles within treatments (in light and in dark).


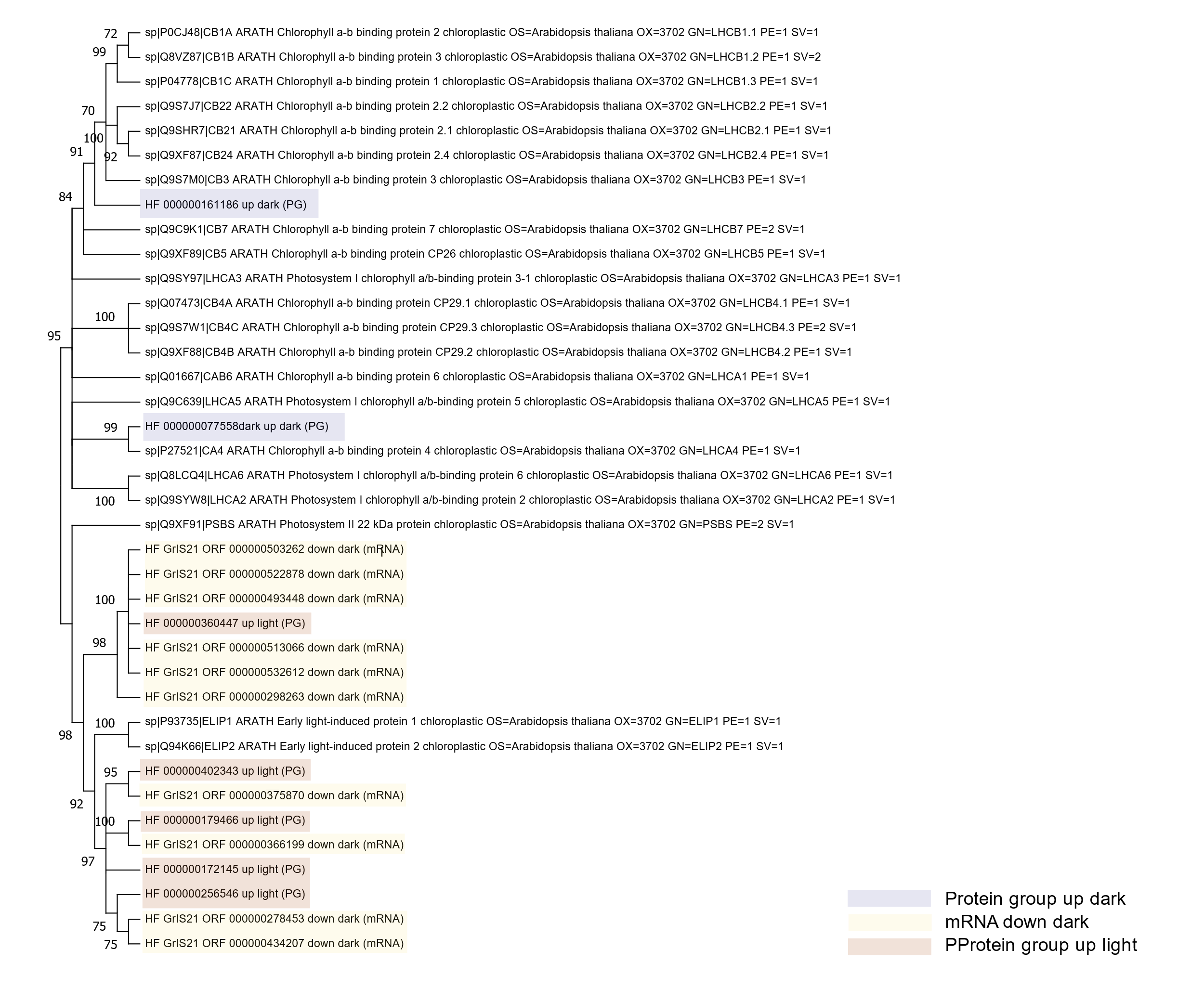


**Supplementary Figure S13: Maximum likelihood tree with 100 bootstraps of different LHC protein sequences**. Includes sequences in our study, and all Arabidopsis thaliana protein sequences with a Chlorophyll A-B binding protein domain (retrieved from UNIPROT). The tree is condensed with a bootstrap cut-off value of 70.
